# Neural Influences on Tumor Progression Within the Central Nervous System

**DOI:** 10.1111/cns.70097

**Published:** 2024-10-29

**Authors:** Wenhao Lv, Yongjie Wang

**Affiliations:** ^1^ Affiliated Hospital of Hangzhou Normal University Hangzhou Normal University Hangzhou Zhejiang China; ^2^ School of Pharmacy Hangzhou Normal University Hangzhou Zhejiang China

**Keywords:** central nervous system tumors, neuron, oligodendrocyte precursor cell, paracrine signal, synapse

## Abstract

For decades, researchers have studied how brain tumors, the immune system, and drugs interact. With the advances in cancer neuroscience, which centers on defining and therapeutically targeting nervous system‐cancer interactions, both within the local tumor microenvironment (TME) and on a systemic level, the subtle relationship between neurons and tumors in the central nervous system (CNS) has been deeply studied. Neurons, as the executors of brain functional activities, have been shown to significantly influence the emergence and development of brain tumors, including both primary and metastatic tumors. They engage with tumor cells via chemical or electrical synapses, directly regulating tumors or via intricate coupling networks, and also contribute to the TME through paracrine signaling, secreting proteins that exert regulatory effects. For instance, in a study involving a mouse model of glioblastoma, the authors observed a 42% increase in tumor volume when neuronal activity was stimulated, compared to controls (*p* < 0.01), indicating a direct correlation between neural activity and tumor growth. These thought‐provoking results offer promising new strategies for brain tumor therapies, highlighting the potential of neuronal modulation to curb tumor progression. Future strategies may focus on developing drugs to inhibit or neutralize proteins and other bioactive substances secreted by neurons, break synaptic connections and interactions between infiltrating cells and tumor cells, as well as disrupt electrical coupling within glioma cell networks. By harnessing the insights gained from this research, we aspire to usher in a new era of brain tumor therapies that are both more potent and precise.

## Graphical abstract

Neurons can establish connections with tumor cells via chemical or electrical synapses, regulating the progression of tumors in the central nervous system. In addition, they can also enhance tumor proliferation and the formation of neurogliomal synapses by secreting proteins into the tumor microenvironment through paracrine signaling.

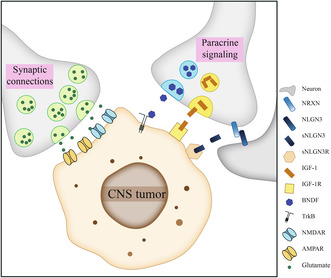

**Abbreviations**
ADAM10: A‐disintegrin‐and‐metalloprotease 10
AMPA: α‐amino‐3‐hydroxy‐5‐methyl‐4‐isoxazole propionic acid
BDNF: brain‐derived neurotrophic factor
CaMK: calmodulin‐dependent kinase
CCL2: CC chemokine ligand 2
CCL7: CC chemokine ligand 7
CNS: central nervous system
CPNs: callosum projection neurons
CTCs: circulating tumor cells
DTCs: disseminated tumor cells
EGFR: epidermal growth factor receptor
EPSPs: excitatory postsynaptic potentials
ER: estrogen receptor
ERK: extracellular regulated protein kinases
FGFR: fibroblast growth factor receptor
GBM: glioblastoma multiforme
GDNF: glial cell line‐derived neurotrophic factor
GJs: gap junctions
HFC: high functional connectivity
HGF: hepatocyte growth factor
HGGs: high‐grade gliomas
ICWs: intercellular calcium waves
IGF‐1: insulin‐like growth factor‐1
LBC: luminal breast cancer
LTP: long‐term potentiation
M/T: mitral and tufted
MAPK: mitogen‐activated protein kinase
MEK‐MAPK: extracellular signal‐regulated kinase‐mitogen‐activated protein kinase
MICs: metastasis‐initiating cells
MMPs: matrix metalloproteinases
NBTSC: neuron‐to‐brain tumor synaptic communication
NF: nuclear factor
NF‐κB: nuclear factor kappa‐B
NGS: neurogliomal synapses
NLGN3: neuroligin‐3
NMDAR: N‐methyl‐d‐aspartate receptor
NRXN: neurexin
NRXN1b: neurexin‐1b
OPCs: oligodendrocyte precursor cells
PCs: prohormone convertases
pHGGs: pediatric high‐grade gliomas
PI3K‐mTOR: phosphoinositide 3‐kinase/mammalian target of rapamycin
proBDNF: precursor of BDNF
PSD: postsynaptic density
PSPs: postsynaptic potentials
SCLCs: small‐cell lung cancer cells
SDI: social population indices
sNLGN3: secretory NLGN3
TAMs: tumor‐associated macrophages
TME: tumor microenvironment
TMs: tumor microtubules
Tregs: regulatory T cells
VEGF: vascular endothelial growth factor

## 1 Introduction

Brain tumors refer to a diverse group of tumors that can originate from different cells in the central nervous system (CNS) or from systemic cancers that metastasize to the CNS [1, 2, 3, 4, 5]. Primary brain tumors encompass a range of histological subtypes, with the most prevalent being gliomas, meningiomas, pituitary adenomas, and acoustic neuromas. Among systemic cancers, lung, melanoma, and breast cancers are particularly prone to CNS metastasis [4]. These tumors can manifest symptoms and signs by invading local brain tissue, compressing adjacent structures, and elevating intracranial pressure. Additionally, the clinical presentation of brain tumors is influenced not only by the tumor's histological type but also by the function of the brain area it affects [1, 2, 3, 5]. In the United States, the age‐adjusted incidence of primary brain and nervous system tumors is about 25 cases per 100,000 individuals, with roughly 30% being malignant [6]. Globally, the incidence of brain cancers is on the rise, especially in countries with lower and moderate social population indices (SDI) [7], emphasizing the urgent need for effective treatment strategies.

The nervous system plays a pivotal role in regulating health. It is composed of a variety of neurons and glial cells with diverse functions and morphologies. While the nervous system facilitates reflexes and nerve conduction, enabling adaptive responses to environmental stimuli, it also orchestrates organ development, maintains homeostasis, and regulates tissue regeneration—all vital processes that underpin the functioning of organ systems, including the immune and endocrine systems [8, 9, 10, 11, 12, 13, 14, 15, 16]. Within the CNS, neurons and glial cells engage in intricate, yet complementary roles, with astrocytes nourishing neurons, oligodendrocytes enhancing signal transmission, and microglia acting as the immune sentinels, protecting neurons, and modulating neural plasticity [17, 18, 19, 20, 21, 22]. The dynamic interplay between neurons and glial cells is crucial for preserving CNS stability, exerting biological impacts, and enabling a range of functions, including human neural reflexes.

Beyond its physiological roles, the CNS also plays a pivotal regulatory part in pathological states. The emergence of cancer neuroscience has provided a novel perspective for cancer and nervous system research [23, 24]. Cancer neuroscience, as a research field, can be traced back to the 19th century when Young et al. discovered an abundance of nerve fibers in tumors, which became the first evidence of neural involvement in tumor progression [25]. Scherer et al. further contributed by uncovering the “satellite phenomenon,” where glial cells were observed surrounding nerve cells within intracranial gliomas, providing preliminary evidence for the important role of nerves in the occurrence and development of malignant tumors [25]. In 2001, Ayala and colleagues presented in vitro evidence that interaction between nerves and prostate cancer cells can affect cancer growth [26]. These foundational studies laid the groundwork for the evolution of cancer neuroscience and paved the way for future research and clinical explorations. In 2015, a milestone was achieved when a team led by Professor Michelle Monje from Stanford University identified the driver gene Neurolign‐3 (NLGN3), establishing a link between cortical neuron activity and the proliferation of high‐grade gliomas (HGGs), thereby illustrating the influence of neuronal activity on brain tumor growth [27]. The following years saw a significant breakthrough in 2019, with concurrent publications in *Nature* by Professor Monje's team at Stanford University, Thomas Kuner's at Heidelberg University, and Douglas Hanahan's at the Swiss Federal Institute of Technology in Lausanne. These papers collectively revealed that brain tumor cells can form excitatory synapses with neurons, a mechanism that fosters tumor growth [28, 29, 30]. This discovery further elucidated the intricate relationship between excitatory synapses, glial cells, and their role in promoting tumor invasion and proliferation within the tumor microenvironment. Culminating in December 2019, a symposium at the Cold Spring Harbor Banbury Center convened 35 experts from diverse fields including cancer, neuroscience, immunology, and developmental biology. This gathering aimed to chart a course for the burgeoning field of cancer neuroscience. Their collaborative efforts culminated in a comprehensive review published in *Cell* on April 16, 2020, delineating the contours of this emerging discipline and heralding a new era in cancer neuroscience [23]. Nowadays, the focus is not solely on studying neurons or tumor cells in isolation. Instead, there is a growing consensus that the nervous system and tumor can interact at both local and systemic levels. Neurons and glial cells not only directly communicate with tumor cells but also remotely influence immune responses [23, 24, 31, 32, 33]. Synaptic communication between neurons and brain tumor cells can regulate tumor growth through neurotransmitters and voltage regulation mechanisms, paracrine substances between neurons and tumor cells facilitate signal transmission between them, substances secreted by neoplastic tissue can affect nervous system function, and tumor treatment also causes neurological toxicity, ranging from peripheral neuropathy to cognitive impairment [23, 24, 31, 32, 33].

The interaction between neurons and tumor cells plays an important role in the development of cancer, especially in the regulation of the tumor microenvironment (TME). The TME is a complex ecosystem comprising not only tumor cells but also non‐malignant cells, extracellular matrix, blood vessels, lymphatic vessels, nerve fibers, and various intercellular communication molecules. As interdisciplinary research at the nexus of neuroscience and cancer biology advances, the intricate link between the nervous system and oncogenesis has become increasingly evident. These dynamic relationships can promote the growth, invasion, and evasion of immune surveillance of tumors through diverse mechanisms [3, 24, 25, 32, 33, 34, 35, 36, 37, 38, 39, 40, 41, 42]. Neuronal activity can directly affect tumor cells through electrical and chemical signals [28, 29, 30, 43, 44, 45]. For example, optogenetic stimulation has been shown to trigger tumor proliferation in specific neural circuits, underscoring the significant role of neural activity in the proliferation of HGGs and the occurrence of low‐grade gliomas [27, 43]. Additionally, neuronal activity can affect the behavior of tumor cells by releasing proteins such as brain‐derived neurotrophic factor (BDNF), NLGN3, and insulin‐like growth factor‐1 (IGF‐1) in the microenvironment. These factors can promote the proliferation, survival, and invasion of tumor cells [44, 45, 46]. Synaptic formation between neurons and tumor cells facilitates communication, with glutamatergic synapses, particularly those mediated by α‐amino‐3‐hydroxy‐5‐methyl‐4‐isoxazolepropionic acid (AMPA) receptors, playing a pivotal role in the TME [29, 30, 31, 44].

Furthermore, glioma activity can indirectly shape the TME by modulating the recruitment and activation of immune cells. Secretion of cytokine and chemokine gradients released by GBM, like CC chemokine ligand 2 (CCL2) and CCL7, glial cell line‐derived neurotrophic factor (GDNF), hepatocyte growth factor (HGF), and so on [47, 48], induces entry of immune cells to tumor microenvironment, thus influencing glioma‐associated microglia/macrophages and fostering tumor maintenance and progression. Glial cells within the glioma microenvironment, including microglia and astrocytes, display notable heterogeneity and can regulate the tumor's immune contexture via multiple pathways, such as cytokines, signaling pathways, immune checkpoints, and chemokines. The glioma microenvironment often exerts a potent immunosuppressive effect, with tumor‐associated macrophages (TAMs) and microglia critically involved in sculpting this suppressive milieu. These cells can suppress immune responses through various mechanisms, including inhibiting T cell activation and promoting the infiltration of regulatory T cells (Tregs) [34, 49, 50, 51, 52, 53, 54, 55, 56, 57, 58].

On the contrary, tumor cells can reciprocally affect neurons. Glioma cells can release neurotransmitters, such as glutamate, which can disrupt neuronal activity, potentially precipitating conditions like epilepsy [59]. Moreover, gliomas can impair cognitive function and influence patient survival by disrupting the formation of brain functional network circuits [60, 61, 62]. GBM can promote its own growth through some molecules, but the transfer of this proliferation signal to neurons can lead to neurodegeneration. For example, miR‐26 can drive the GBM cycle progression, and an increase in miR‐26 levels can cause abnormal entry of neurons into the cell cycle after mitosis, leading to neuronal cell death. The intercellular transfer of such molecules from glioblastoma to neurons may affect neuronal health, post mitotic status, and overall cell vitality [34, 63, 64, 65, 66]. This bidirectional communication between the nervous system and tumor cells underscores the complexity of interactions within the TME and highlights the need for targeted therapeutic strategies that address these multifaceted relationships.

In this review, we delved into the complex dynamics between neurons and CNS tumors—encompassing both primary intracranial growths and those that have metastasized to the brain. We elucidated the role of neurons in the progression of CNS tumors from the perspectives of synaptic structure, paracrine signaling, and the evolution of tumor precursor cells. We also provided a comprehensive overview of the reciprocal effects these entities exert on one another, which can contribute to help developing innovative and effective treatment methods with interdisciplinary collaboration.

## 2 Synaptic Connections Between Neurons and Brain Tumor Cells

Synapse refers to the structure in which impulses from one neuron are transmitted to another neuron or another cell through mutual contact [67, 68, 69]. In the human brain, synapses are divided into two major categories based on their structure and signal transmission methods: electrical and chemical synapses. Traditionally, people pay more attention to the role of synapses in functional interaction and information transmission among neurons [67, 68, 69, 70].

In recent years, mounting studies have uncovered the significant influence synapses exert on the progression and behavior of tumors. Neurons have been observed to form direct synaptic connections with tumor cells and may directly influence them [44, 45]. Of particular interest is the discovery of tripartite synapses, which consist of presynaptic and postsynaptic neurons along with encroaching tumor cells [30, 44, 71]. These tripartite configurations enable neurons to modulate tumor cells through both electrical signaling and the release of neurotransmitters [30, 44].

### 2.1 Chemical Synapses

Chemical synapses rely on the release of specific chemicals from the terminals of presynaptic neurons as a medium for transmitting information to affect postsynaptic neurons [72, 73, 74]. In the context of CNS tumor, neuron‐to‐tumor chemical synapses consist of a presynaptic neuron and a postsynaptic tumor cell [28]. As a key structure for neural signal transmission, it is reasonable to hypothesize that tumor cells may “hijack” or “deceive” neurons into forming synaptic or pseudo‐synaptic structures and potentially harnessing this ability to trigger neural plasticity to enable gliomas to receive additional neuronal signals and rapidly proliferate [44]. However, not all tumor cells are capable of forming synapses with neurons. For example, in neurogliomal synapses (NGS), these connections occur exclusively between higher‐grade gliomas and neurons [28].

This selectivity may stem from the heterogeneity of brain tumors, with tumor cells originating from various brain cell types, including glial cells, precursor cells of glial cells, and metastatic tumors. Incompatibility in molecular and structural profiles between tumor and neuronal cells could preclude synapse formation. Moreover, the gene or protein expression profiles vary among different tumors, and even within the same type, which may affect the interaction between tumor cells and neurons; It was found that a large number of genes involved in neural circuit assembly or remodeling were upregulated in the high functional connectivity (HFC) tumor region [61]. A case in point is thrombospondin‐1 (TSP‐1), which plays a role in synaptic formation and is predominantly secreted by astrocytes, with its expression elevated seven‐fold in HFC areas [61, 75, 76]. Compared with the low functional connectivity (LFC) region, the synaptic markers (Synapsin and PSD‐95) in HFC are increased, indicating enhanced synaptic stability and formation in HFC of glioblastoma multiforme (GBM) [61].

**FIGURE 1:**
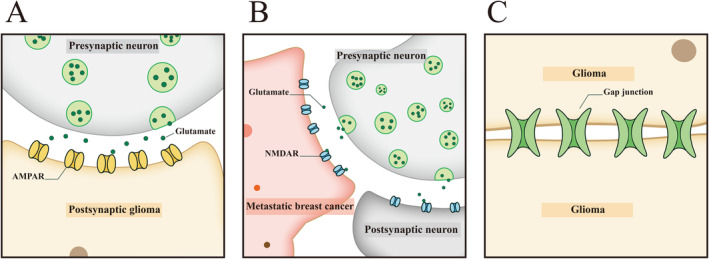
Synaptic connections between neurons and brain tumor cells and their implications in tumor progression. (A) The involvement of AMPA receptors in glioma biology is depicted. Neuronal activity initiates the opening of calcium‐permeable AMPA receptors, which are essential for mediating the electrophysiological functions of neurons. The activation of these receptors allows for the transmission of signals to synapses with glioma cells, leading to the depolarization of the glioma cell membranes. This depolarization is a critical event that promotes the proliferation of tumor cells. The diagram illustrates the process where the presynaptic neuron releases glutamate, which then binds to the AMPA receptors on the postsynaptic glioma cell, initiating a series of intracellular signaling events that result in tumor growth. (B) The formation of aberrant synaptic connections between neurons and breast cancer cells in the brain is shown. These “triple synapses” involve presynaptic neurons, postsynaptic tumor cells, and the release of neurotransmitters like glutamate. The interaction between glutamate and NMDA receptors on the cancer cells' membrane triggers a cascade of events that intensify the metastasis and proliferation of breast cancer cells within the brain. The diagram highlights the unique synaptic structure where the breast cancer cells release glutamate, which in turn activates NMDA receptors, fostering continuous synaptic transmission and contributing to tumor progression. (C) The role of gap junctions in the intercellular communication between glioma cells is detailed. Glioma cells establish extensive networks primarily through the formation of gap junctions, which are conduits for the exchange of signaling molecules, including calcium ions, between adjacent glioma cells. This exchange is instrumental in influencing the migration and proliferation of tumor cells, indicating the significance of gap junctions in the collective behavior of glioma cell populations. The diagram illustrates how these gap junctions extend into the surrounding tissues, enhancing the invasiveness and proliferation of brain tumors and facilitating the communication that drives tumor growth. These synaptic connections and networks represent potential therapeutic targets for disrupting the supportive role of neurons in brain tumor progression, offering new avenues for the development of treatments aimed at modulating these interactions to inhibit tumor growth and spread.

In addition, immunoelectron microscopy studies have revealed that the BDNF/TrkB signaling pathway can increase the number of synaptic connections between neurons and glioma cells [44]. Mechanistically, BDNF, upon binding to TrkB, escalates the transport of α‐amino‐3‐hydroxy‐5‐methyl‐4‐isoxazole propionic acid (AMPA) receptors (AMPAR) to the glioma cell membrane, increases calcium ion flux, intensifies and prolongs electrical signaling, and subsequently boosts the depolarization amplitude of glioma cell membranes, ultimately promoting glioma cell mitosis [44, 77, 78]. Notably, the synapses between neurons and tumor cells are predominantly glutamatergic [28, 79], with most NGS composed of AMPAR (Figure 1A) [28, 80, 81]. Typically, NGS contain a neurogenic presynaptic membrane, a synaptic cleft with electron‐dense material, a neoplastic postsynaptic membrane zone matrix with docked vesicles, and a postsynaptic density area [28, 82]. The influence of neurons on glioma is multifaceted through NGS [28, 29, 82]. Firstly, NGS can activate glioma networks. Glioma cells within the brain are not isolated entities [83]. They form extensive channels, known as tumor microtubules (TMs), through their cell membranes. These TMs link multiple tumor cells, enabling them to couple and communicate via gap junctions [28, 84, 85]. After the vesicles from presynaptic neurons fuse with the anterior membrane, glutamate is released, crosses the synaptic cleft, and binds to the AMPA receptor on the postsynaptic membrane [80, 81]. After the AMPA receptor is excited, a large amount of ions will flow inward to produce excitatory postsynaptic potentials (EPSPs) [29]. The glioma cell subpopulation stimulated by synapses can transmit calcium waves to the remaining TM connected glioma network, enhancing some inimical attributes of gliomas [28, 29, 83]. Secondly, neural activity can drive glioma invasion and proliferation through NGS [28]. After the activation of AMPA receptor, it will cause transmembrane ion flow, depolarization, and excitation of glioma [28]. Inhibiting AMPA receptors or gap junctions in gliomas could impede their growth, whereas enhancing AMPA receptor signaling could expedite progression [28, 29]. Further exploration is required to uncover the precise mechanisms driving glioma advancement.

The N‐methyl‐d‐aspartate receptor (NMDAR) represents a distinct subtype of glutamatergic synapses, as depicted in Figure 1B [86, 87]. This neuron‐to‐tumor subtype is identified on the postsynaptic membrane of synapses formed by brain metastatic breast cancer cells and neurons [30]. In a divergence from typical synaptic structures, these breast cancer cells engage in the formation of “pseudo tripartite synapses” with neurons, which are known to release glutamate as a neurotransmitter. Such synapses are pivotal to the metastatic process in the brain and are correlated with a poor prognosis. They are similar to the tripartite synapses formed between two neurons and surrounding non‐neuronal supporting cells such as astrocytes [71]. Once these synapses are established, neurons incessantly supply glutamate to the breast cancer cells, facilitating a continuous synaptic transmission [30, 88]. The interaction between glutamate and NMDARs on the cancer cells' membrane triggers depolarization, thereby intensifying the metastasis and proliferation of breast cancer cells within the brain [30, 71, 88]. Studies also have found that breast cancer cells activate NMDARs by the autocrine secretion of glutamate and then promote cell proliferation and migration [30]. Utilizing an immunodeficient mouse model, research has demonstrated that the deletion of these NMDARs markedly reduced the colonization and growth of injected human B2BM breast cancer cells in the brain [30]. The expression of NMDAR seems to be directly related to brain metastasis of breast cancer, although the precise mechanism for this is yet to be elucidated.

The process of brain metastasis in breast cancer is conceptualized as a two‐step phenomenon. Initially, cancer cells must successfully colonize the organ, a process that is considered the primary rate‐limiting step in metastatic spread [89, 90, 91, 92]. Subsequently, the establishment of tripartite synapses occurs, which is crucial for the progression of metastasis. Research indicates that the NMDAR, along with its downstream signaling effectors MEK‐MAPK and CaMK, when activated by self‐secreted glutamate, can enhance the invasive capabilities of breast cancer cells [93, 94].

Within the central nervous system, glutamate serves as the predominant excitatory neurotransmitter, being particularly concentrated in the brain. This abundance may account for the propensity of breast cancer cells to metastasize to the brain. NMDARs in neuronal cells are instrumental in synapse formation and function, initiating various signaling pathways like CaMKII and PKC through Ca^2+^ influx [95, 96, 97, 98, 99, 100]. Moreover, NMDARs interact with a variety of proteins at the postsynaptic density (PSD), including PSD‐95, SynGAP, and Shank. These interactions are essential for receptor stabilization on the postsynaptic membrane and for modulating synaptic maturation and plasticity by creating a complex protein network [101, 102, 103, 104, 105, 106, 107, 108, 109, 110].

It is hypothesized that NMDARs in breast cancer cells may exploit glutamate released by neurons to facilitate the formation of their own synaptic structures. Within the tripartite synaptic framework, these cancer cells could secure a steady supply of glutamate, analogous to parasitic relationships. Furthermore, evidence suggests that breast cancer cells which have metastasized to the brain are subject to influences from the brain's microenvironment. This can induce a reprogramming of the cells, mirroring the neurogenesis that occurs during developmental stages [111, 112]. This insight underscores the complexity of the metastatic process and points to potential targets for therapeutic intervention.

It is noteworthy that while normal breast cells typically do not express NMDARs, in instances of breast cancer, particularly when it metastasizes to the brain, the cancer cells may begin to express these receptors. The acquisition of NMDARs by breast cancer cells could involve a range of biological processes, including genetic mutations, genomic amplifications, epigenetic modifications, and body weight sorting. It is particularly intriguing that NMDARs, predominantly expressed in neurons, are also expressed by tumors that have metastasized to the brain from other parts of the body [30, 113]. For example, small‐cell lung cancer cells (SCLCs) with brain metastases secrete Reelin, a brain development factor specifically produced by Cajal–Retzius cells in the marginal zones of the cerebral cortex and hippocampus [114, 115]. This secretion attracts astrocytes to the brain metastases [113], which in turn promote the growth of SCLCs by releasing neuronal survival factors, such as SERPINE1 [113].

Recent research has found that the axons of corpus callosum projection neurons (CPNs), namely glutamatergic excitatory neurons, can traverse the cortical hemisphere along the corpus callosum, thereby driving the progression of contralateral GBM [116].

### 2.2 Gap Junction

Here, we will further discuss the communicated connections formed among tumor cells (Figure 1C) from the perspective of cancer neuroscience and discuss electrical signals mediated by ion flow. Gap junctions (GJs), well‐established conduits for direct intercellular communication, are prevalent in the CNS and convey both chemical and electrical messages among neurons [117]. Beyond their recognized roles in processes like metabolic regulation, ion buffering, and energy transfer, GJs also play a part in orchestrating calcium waves, ATP receptor signaling, neural development, and sustaining the nervous system's distinctive functions [84, 117, 118, 119, 120, 121, 122]. The primary modality of communication and connection identified between brain tumor cells to date is through GJs, yet the potential molecular signals and additional connecting structures are subjects of ongoing investigation [28, 29, 30, 83, 84, 118]. GJs seem to be highly utilized by gliomas as an important component of the glioma network. Studies have revealed that the synapses that bridge neurons and tumors, formed within the TME, possess electrophysiological functions [28, 29, 30, 84]. As previously discussed, glioma cells possess exceptionally elongated tubular extensions on their cell membranes, termed “tumor microtubes.” These structures enable the formation of interconnected, multicellular functional networks of glioma cells via GJs [123].

Multiple models of orthotopic xenotransplantation from different patient sources have been used in research [29]. A case in point is the stereotactic injection of GFP‐tagged glioma cells into the CA1 region of the mouse hippocampal circuit. Following adequate transplantation and growth, whole‐cell patch clamp recordings were conducted on GFP‐positive glioma cells within acute hippocampal slices. The results demonstrated that the excitatory postsynaptic currents (EPSCs) of glioma cells depend on neuronal action potentials, with synaptic transmission being carried out via AMPA receptors [29]. Thus, this electrophysiological data corroborate the presence of genuine synapses between neurons and gliomas, capable of inducing electrical excitation in glioma cells through these synaptic connections [29].

Neuron activity enhances glioma excitation by modulating the secretion of paracrine growth factors and facilitating electrochemical communication through synapses between neurons and glioma cells [27, 43, 45, 46]. Concurrently, the influx of calcium ions triggers cell communication within the glioma, propagating activation signals via intercellular calcium waves (ICWs) to other glioma cells through TMs [28, 29, 80]. Currently, a large amount of evidence suggests that neurons and glioma cells, including GBM occurred in both adults and children [29, 44, 84, 124], are directly connected via GJ on the surface of TMs. Here, the AMPA subtype of glutamate receptors relays postsynaptic electrical signals, prompting tumor cells to infiltrate and proliferate autonomously [28, 29, 30]. Recent studies have identified that Ca^2+^ specifically activates the MAPK and NF‐κB signaling pathways within cellular networks, ultimately driving brain tumor growth [123]. Additionally, neuronal activity also evokes non‐synaptic activity‐dependent potassium currents that are amplified through gap junction‐mediated tumor interconnections by forming an electrically‐coupled network [29, 83, 84]. The elevation of extracellular potassium concentration, a consequence of neuronal action potentials, augments neuronal excitability within the glioma microenvironment. This increase enhances the duration of potassium currents in non‐synaptic glioma and intensifies the excitability of synaptic neuron‐to‐glioma EPSCs [29].

As has been said before, the interconnected network of tumor cells, called “glioma network” and underpinned by this structural framework and the electrical signals conveyed through gap junctions, appears to be pivotal to glioma growth [28, 29, 84, 85, 123, 125, 126, 127].

## 3 Paracrine Signals From Neurons in the Occurrence and Growth of Brain Tumors

As our understanding of the brain deepens, the pivotal role of the TME in CNS tumors has come into sharper focus [50, 56]. The TME encompasses the internal and external conditions surrounding tumor cells that are intricately linked to tumor occurrence, growth, and metastasis [128]. Within this context, neurons are a crucial component of the TME for malignant brain tumors, exerting biological influences by releasing various factors into the TME [128]. Notably, both brain‐derived neurotrophic factor (BDNF) and synaptic protein NLGN3 are instrumental in the proliferation of glioma through paracrine secretion in the TME. Furthermore, insulin‐like growth factor‐1 (IGF‐1) has been identified as a key paracrine regulator of neuronal activity, propelling the growth of olfactory gliomas [27, 43, 45, 46].

### 3.1 NLGN3

**FIGURE 2:**
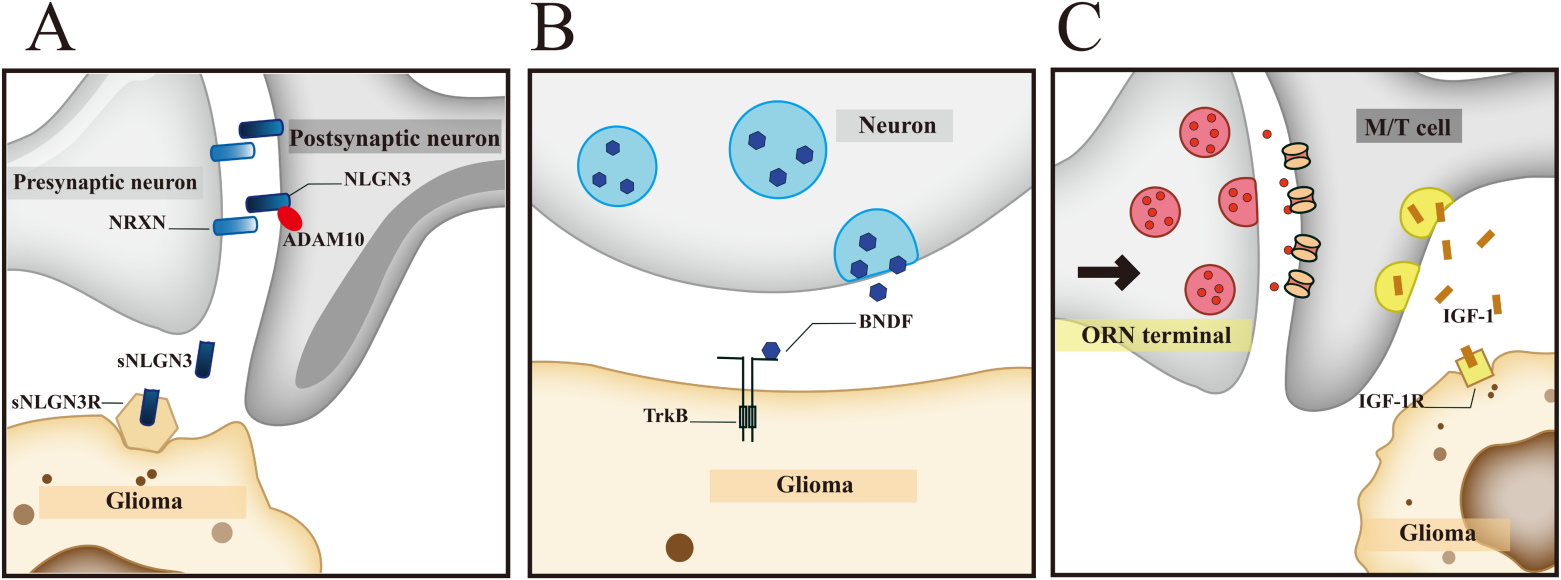
Paracrine signals from neurons in the occurrence and growth of brain tumors. (A) The role of the synaptic protein neuroligin‐3 (NLGN3) in the tumor microenvironment (TME) is illustrated. The secretory form of NLGN3, generated by the action of the protease ADAM10, is depicted as a key factor that stimulates the proliferation of glioma cells. This process underscores the contribution of synaptic proteins to the complex interactions within the TME that can promote glioma growth. The diagram shows how NLGN3, once cleaved and released, can bind to its receptors on glioma cells, initiating downstream signaling pathways that enhance cell survival and proliferation. (B) The BDNF/TrkB signaling pathway and its implications for synaptic plasticity and tumor malignancy are detailed. When dysregulated, this pathway can augment the complexity and strength of the tumor's synaptic network, thus fostering further tumor progression. The diagram illustrates the mechanism by which BDNF, secreted by neurons, interacts with the TrkB receptor on tumor cells, initiating a cascade of intracellular signaling events that can lead to increased tumor cell survival, growth, and potentially the formation of stronger synaptic connections with neurons. (C) The influence of olfactory neurons on the proliferation of oligodendrocyte precursor cells (OPCs) through the release of insulin‐like growth factor 1 (IGF1) is shown. Specifically, the mitral and tufted (M/T) cells in the olfactory bulb are highlighted as the primary source of IGF1, which can accelerate the proliferation of OPCs that have undergone pro‐oncogenic mutations. The diagram depicts the process where sensory input, such as the presence of certain gases, stimulates olfactory neurons, leading to the release of IGF1 and the subsequent promotion of gliomagenesis in the olfactory bulb. These paracrine signals represent critical mechanisms by which neurons can modulate the behavior of tumor cells in the brain. Understanding these pathways is essential for developing targeted therapies that may disrupt the supportive role of the neuronal environment in brain tumor growth and spread.

NLGN3, a vital nerve ligand and cell adhesion protein located on the postsynaptic membrane, is integral to the formation and upkeep of synapses between neurons, thereby sustaining proper neural functions [27, 129]. Historically, NLGN3 has garnered significant attention due to its pivotal role in autism spectrum disorders [130, 131]. A vital mechanism that orchestrates the neural regulation of brain cancer is the activity‐dependent cleavage and secretion of NLGN3, which promotes glioma proliferation through multiple signaling pathways (Figure 2A) [27, 45, 132]. NLGN3 connects to presynaptic neurons via Neurexin (NRXN) located on the presynaptic membrane [129, 133]. A‐disintegrin‐and‐metalloprotease 10 (ADAM10), a member of the metzincin metalloproteases crucial for intercellular communication by modulating membrane protein functions, cleaves NLGN3, enabling it to manifest biological effects [134, 135]. This cleavage transforms NLGN3 into its secretory form (sNLGN3), which subsequently acts on glioma cells within the TME to promote tumor progression [27, 45, 136].

The most classical downstream pathway of NLGN3 is the phosphoinositide 3‐kinase/mammalian target of rapamycin (PI3K‐mTOR) pathway [27, 45, 136]. Emerging studies have illuminated that NLGN3 acts on glioma cells accompanied by activation of the extracellular regulated protein kinases (ERK) pathway and nuclear factor kappa‐B (NF‐κB) pathway [45, 136]. In previous studies, activation of the PI3K‐mTOR pathway can inhibit apoptosis induced by various stimuli, promote cell cycle progression, and thus enhance cell proliferation and survival [137, 138]. It also contributes to the formation of tumor vasculature and plays a cardinal role in tumor metastasis and development [27, 139, 140]. Within the ERK pathway, through the three‐stage kinase cascade reaction of mitogen‐activated protein kinase (MAPK) signal transduction, phosphorylated ERK1/2 is translocated from the cytoplasm to the nucleus. There, it mediates the transcriptional activation of *Elk‐1*, *ATF*, *Ap‐1*, *c‐fos*, and *c‐Jun*, and results in cell proliferation and differentiation [45, 136, 141, 142].

Recent experimental evidence indicates that the dual knockout of Gαi1/3 significantly represses the expression of NLGN3, subsequently inhibiting mTORC1 and Erk activation, which are downstream of NLGN3. This inhibition effectively curbs the proliferation and migration of glioma cells [136]. Additionally, NLGN3 is crucial for the establishment of NGS through the PI3K‐mTOR signaling pathway [27, 45]. When neurons are activated, they secrete considerable levels of NLGN3 into the TME, where it interacts with presynaptic membrane proteins, facilitating synaptic maturation and preserving neuronal function. Furthermore, NLGN3 has been shown to phosphorylate key receptors on tumor cells, including VEGF, epidermal growth factor receptor (EGFR), fibroblast growth factor receptor (FGFR), and integrins, thus exerting their biological effects [27, 45, 143].

Interestingly, the NF‐κB signaling pathway is activated in both NLGN3‐mediated effects and within the glioma network [45, 123]. The activation of this pathway has been extensively validated in glioma research, highlighting its pivotal role in tumorigenesis. The growth of tumor cells and tissue invasion require continuous neovascularization, of which proteins are affected by NF‐κB adjustment [144]. Among them, vascular endothelial growth factor (VEGF), the most important member of the angiogenic factor family, and nuclear factor (NF), which is continuously activated by NF‐κB, can enhance the transcription of *VEGF* [145]. Furthermore, NF‐κB can significantly inhibit the transcription of apoptosis‐related genes, such as *c‐IAP1/c‐IAP2* [146], tumor necrosis factor receptor binding factor *TRAF1/TRAF2* [147], and zinc finger protein *A20* [144, 148, 149]. The two characteristic stages of malignancy development are tissue invasion and metastasis, which can also be caused by the regulation of NF‐κB‐dependent genes, including matrix metalloproteinases (MMPs), urokinase plasminogen activator, interleukin‐8, and so on [144, 148, 149]. Currently, research is scarce, and concrete evidence is lacking regarding the specific effects of the interaction between NLGN3 and glioma by the NF ‐κB pathway, further research is warranted to elucidate these mechanisms.

Previous research has firmly established that higher levels of *NLGN3* mRNA and protein, derived from tumors, are inversely linked to the survival rates of adult glioblastoma patients [150]. The latest findings further suggest a correlation between neuronal secretion of NLGN3 and the severity of gliomas. Specifically, in high‐grade gliomas such as adult glioblastoma, anaplastic oligodendroglioma, pediatric glioblastoma, and diffuse intrinsic pontine glioblastoma (DIPG), increased NLGN3 expression is associated with increased tumor aggressiveness and poorer patient survival [45, 151, 152]. Therefore, understanding the expression patterns of NLGN3 is crucial for improving clinical diagnosis, treatment strategies, and prognostic evaluations.

### 3.2 BDNF

Brain‐derived neurotrophic factor (BDNF) is a crucial protein that facilitates brain plasticity, enabling the strengthening of synaptic connections and the reinforcement of neural circuits formed during learning [153, 154, 155, 156, 157]. In the context of gliomas, these tumors exploit BDNF's mechanisms to their advantage, mimicking the healthy brain's developmental pathways [44]. BDNF, secreted by neurons, moves to tumor cells and initiates intracellular signaling cascades that support tumor growth, ultimately helping the tumor to form more and stronger synapses with neurons [44]. When the cellular mechanisms triggered by BDNF are more strongly activated, tumor cells will respond with stronger currents, which in turn fosters their growth [44]. In other words, tumor utilizes the learning mechanisms of the brain to grow.

BDNF exists in two distinct forms: precursor of BDNF (proBDNF) and mature BDNF [158, 159, 160]. Their distinct functions are carried out through two separate transmembrane receptor signaling systems: p75NTR and TrkB [44, 154, 156, 161]. The roles of the BDNF/TrkB signaling system in tumor cell proliferation and survival have been deeply demonstrated [154, 156, 161]. proBDNF, synthesized by neurons, is then cleaved by prohormone convertases (PCs) and/or furin, or extracellularly by plasmin and MMPs to release the mature homodimeric protein (mature BDNF) outside cells. Mature BDNF activates TrkB receptor with high affinity on glioma surface, thereby promoting cell survival [154, 156, 161]. The activation of the JNK pathway, mediated by BDNF/TrkB signaling, has been implicated in the progression of CNS malignancies [161]. Investigations have substantiated that BDNF serves as a potent activator of signaling cascades such as BDNF/TrkB/PI3K/Akt and TrkB/ATF4, effectively counteracting the inhibitory and apoptotic impacts of BDNF inhibitors on C6 glioma cells [162, 163]. It has been noted in several studies that pediatric gliomas frequently exhibit elevated TrkB expression, a key BDNF receptor, within their malignant cell populations [44, 164]. Further research has demonstrated that genetically or pharmacologically inhibiting TrkB not only negates BDNF's influence on glioma synaptic activity but also significantly enhances the survival rates in xenograft models of pediatric glioblastoma and diffuse pontine glioma [44]. Furthermore, the presence of BDNF and TrkB has also been noted in human gangliogliomas, underscoring their role in glioma biology [165].

While NLGN3 also possesses the capability to stimulate synapse formation between neurons and gliomas [29], its proliferative effect on pediatric cortical high‐grade gliomas (pHGGs) is relatively less pronounced compared to BDNF [45]. This highlights the nuanced roles of various paracrine factors in modulating glioma behavior.

### 3.3 IGF1

In recent years, researchers have made compelling progress in understanding the importance of insulin‐like growth factor (IGF) in the regulation of CNS function. Studies focusing on the pituitary glands of neonatal mice have revealed that IGF‐1 not only bolsters cell survival, growth, and differentiation but also amplifies the neurons' resistance to apoptosis, thereby exerting a neuroprotective influence [166, 167, 168, 169]. In addition, the N‐terminal glycine fragment produced by the hydrolysis of IGF‐1 protein into des‐N‐(1–3) IGF‐1, which is likely the predominant form of IGF‐1 in the brain, has been shown to facilitate neuroprotective effects both in vitro and in vivo [170].

Olfactory gliomas, originating in the olfactory system, predominantly affect the olfactory bulb—the nexus for communication between the primary and secondary neurons of the olfactory circuit [171, 172, 173]. Utilizing a mouse model of primary gliomas that originate from adult oligodendrocyte precursor cells (OPCs) with conditional knockout of the tumor suppressor genes Trp53 and Nf1 (referred to as the CKO model), researchers have observed a high incidence of gliomas in the olfactory bulb, the initial site of olfactory sensory neuron transmission. Additionally, a heightened likelihood of glioma development in other olfactory‐related centers, such as the anterior olfactory nucleus, olfactory nodule, pyriform cortex, and amygdala, has been noted, albeit with a later onset compared to the olfactory bulb.

Detailed anatomical examination of the olfactory bulb's substructures has uncovered that the majority of tumors are confined to the synaptic glomerular layer, the critical site of information exchange between the first‐level neurons (olfactory sensory neurons, ORNs) and the second‐level neurons (mitral and tufted cells, M/T cells) within the olfactory circuit. Notably, IGF1 is predominantly expressed in the mitral and tufted (M/T) cells of the olfactory bulb, but not neurons and glial cells, thereby identifying M/T cells as the principal source of IGF1. Targeted ablation of IGF1 in M/T cells has been demonstrated to effectively curtail the proliferation of mutated oligodendrocytes and to impede tumor progression, emphasizing the critical role of IGF‐1 in glioma development [46].

Previous studies have shown that the function of IGF1 relies on the activation of its receptor, IGF1R. Chronic activation of olfactory receptor neurons in the olfactory bulb after knocking out IGF1R did not promote glioma growth, indicating that olfaction regulates glioma development through the IGF1‐IGF1R signaling pathway (Figure 2C) [46].

This intricate interplay between neurons and glioma cells, mediated by paracrine signals such as BDNF, NLGN3, and IGF‐1, underscores the complexity of the TME in brain tumors and highlights potential therapeutic targets for future interventions.

## 4 The Role of Neurons in Tumor Precursor Cells Should Not Be Underestimated

Within the nervous system, a diverse array of precursor cells possesses the remarkable capacity to differentiate into various cellular constituents, including neurons, astrocytes, and oligodendrocytes, thereby contributing significantly to brain tissue formation. These cells are categorized into several subtypes, such as neural precursor cells, radial glial cells, intermediate progenitor cells, oligodendrocyte precursor cells, retinal precursor cells, and so on [174, 175, 176, 177, 178, 179, 180]. Oligodendrocytes are produced by OPCs [181]. In various types of cancers, the cell sources may be multifunctional neural stem cells, lineage‐restricted neuronal precursor cells, or lineage‐restricted glial precursor cells, each playing a distinct yet complementary role in neural development and function [29, 182, 183, 184, 185].

Primary brain tumors are believed to originate from the neural precursor cell population. HGGs, with their diverse molecular and clinical subtypes, are believed to stem from these precursors, progressing along a differentiation spectrum from less differentiated neural stem cells to more lineage‐restricted OPCs [29, 182, 183, 184, 185].

**FIGURE 3:**
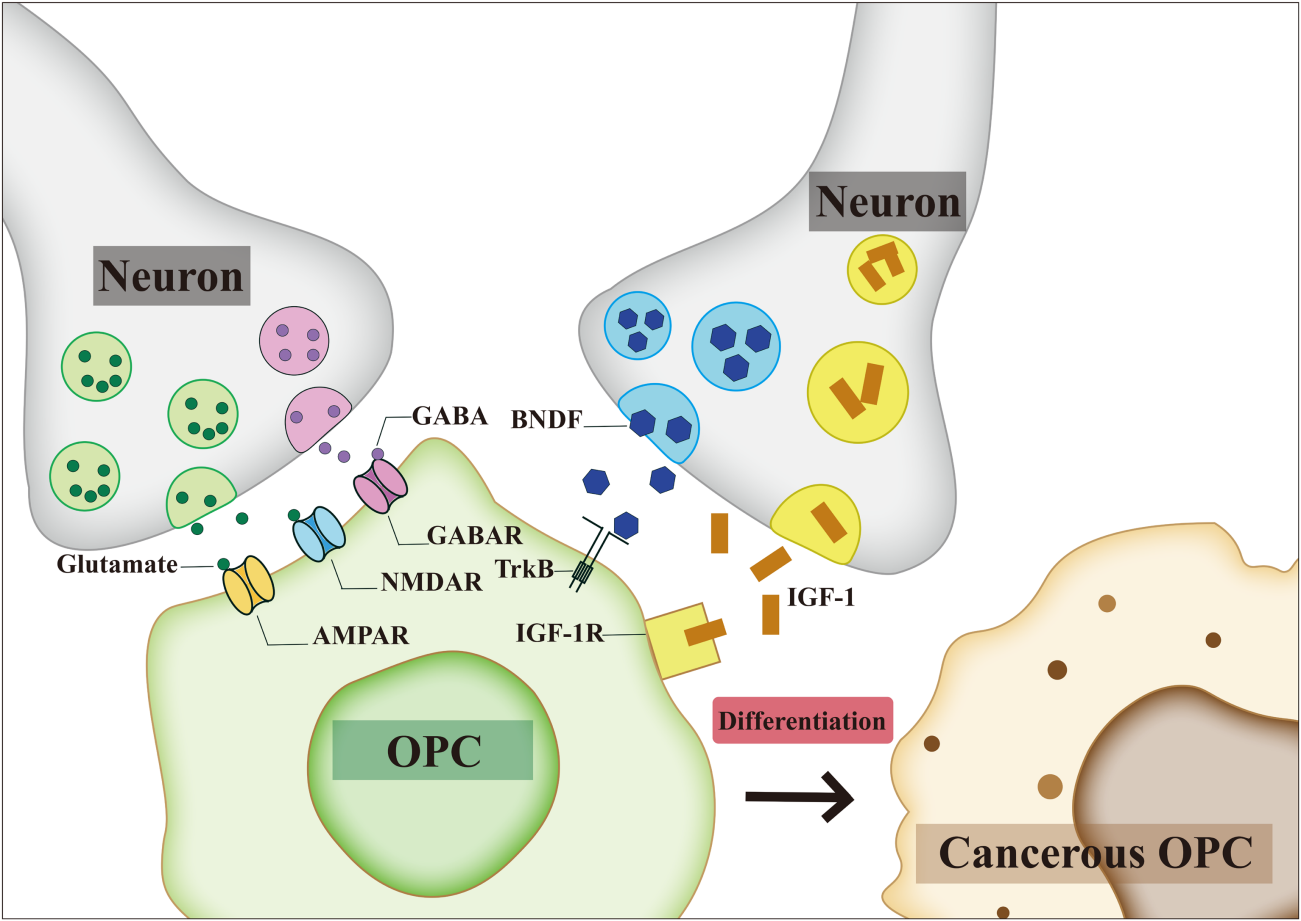
The role of neurons in the transformation of oligodendrocyte precursor cells (OPCs) into tumorigenic cells. The pivotal role of brain‐derived neurotrophic factor (BDNF) and insulin‐like growth factor 1 (IGF‐1) in the transformation of OPCs into glioma cells is highlighted. These factors exert their influence through paracrine signaling pathways, which allow for the modulation of neighboring cells, including OPCs, in a manner that promotes their differentiation into tumorigenic cells. The diagram illustrates how BDNF and IGF‐1, secreted by neurons, bind to their respective receptors on OPCs, triggering intracellular signaling cascades that enhance the survival, proliferation, and potentially the malignant transformation of these cells. The direct synaptic communication between glutamatergic and GABAergic neurons and OPCs is detailed as a crucial aspect of this process. These synaptic interactions facilitate the transmission of electrical and chemical signals that promote the differentiation of OPCs into glioma cells, thereby contributing to the complex interplay between the nervous system and the development of brain tumors. The diagram shows the synaptic connections between neurons and OPCs, indicating the flow of signals that can drive the transformation of OPCs. The potential dysregulation of myelin plasticity that may promote the proliferation of malignant cells within the primary brain cancer group is suggested as an area of future investigation. The diagram represents the hypothesis that the mechanisms involved in myelin development and plasticity could be exploited by malignant counterparts of activity‐responsive neural precursor cells to foster growth. This figure emphasizes the multifaceted communication between neurons and OPCs and how these interactions can lead to the initiation and progression of brain tumors. Understanding these neuronal influences on OPCs is essential for uncovering new therapeutic targets and developing strategies to prevent or treat gliomagenesis.

The connection between neurons and OPCs involves paracrine mechanisms, such as the numerous roles of BDNF in neural development and plasticity, IGF signaling in gliomagenesis, and in addition to promoting tumor proliferation mentioned earlier, also facilitating myelin development [46, 186, 187, 188]. Alternatively, glutamatergic and GABAergic neurons can communicate directly with OPCs through synapses (Figure 3) [43, 79, 189, 190]. Future perspective could involve the dysregulation of myelin plasticity potentially promoting malignant cell proliferation within the primary brain cancer group, or still providing positive feedback on neuronal effects.

Using optogenetic technology to regulate the activity of neurons in the brain, research has found that the activated neurons in cerebral cortex and optic nerve are helpful in promoting the proliferation of OPCs and adapting to myelin sheath changes. The malignant counterparts of these activity‐responsive neural precursor cells may exploit mechanisms of myelin development and plasticity to foster growth [27, 43].

The exploration of primary brain cancer's origins from the neural precursor cell population underscores the profound influence of neuronal interactions and developmental pathways on tumorigenesis. The intricate paracrine and synaptic communications between neurons and OPCs, along with the potential of optogenetic technology to modulate neuronal activity, reveal the critical role of neuronal regulation in both normal brain development and the aberrant proliferation seen in cancer. This understanding not only deepens our insight into the cellular dialogues that drive tumor growth but also opens avenues for innovative therapeutic strategies that target the neuronal mechanisms underlying cancer progression.

## 5 Prospect

### 5.1 Potential Targets and Therapeutic Sites

Elucidating the dynamics of neuron‐brain tumor interactions, particularly the mechanisms by which neurons contribute to tumor progression, opens up new avenues for therapeutic intervention. Existing therapies for CNS tumors encompass a range of treatments, including surgery, radiation therapy, chemotherapy, and targeted therapies [3, 5, 191, 192, 193, 194, 195, 196, 197, 198]. However, due to the unique challenges posed by the blood–brain barrier, the sanctuary of the CNS from immune surveillance, and the heterogeneity of tumor types, traditional therapies have limitations: Surgery, the primary treatment for many CNS tumors, aims to remove as much of the tumor as possible while preserving neurological function, but complete resection is not always feasible, especially for tumors deeply infiltrating critical brain areas [199, 200, 201, 202, 203]; radiation therapy utilizes high‐energy particles or waves to destroy tumor cells which can be delivered in focused doses to the tumor site using techniques like stereotactic radiosurgery, but it can also damage healthy brain tissue [191, 204, 205, 206]; although chemotherapy and targeted therapies can specifically inhibit rapidly dividing cells, the blood–brain barrier often limits the penetration of chemotherapeutic agents into the CNS [3, 5, 193, 194, 197, 198, 207, 208]. Future strategies may focus on two primary approaches: the development of pharmaceuticals to inhibit or neutralize proteins and other bioactive substances secreted by neurons, and the direct delivery of interventional drugs to the synapses formed between neurons and neoplastic cells, or to their downstream targets. Key targets include the inhibition of synaptic and presynaptic signal transmission, the disruption of glutamatergic neuron‐to‐brain tumor synaptic communication (NBTSC), the electrical coupling within glioma cell networks, the synaptogenesis between neurons and glioma, and the hyperexcitability of neurons that stimulates brain tumor growth [24, 209].

The research of cancer neuroscience opens the door to a new avenue of treatment: repurposing neuromodulatory drugs for oncology. Neurotransmitter modulators that affect neurotransmitter release, reuptake, or receptor binding could influence the synaptic connections between neurons and tumor cells. For example, glutamate receptor antagonists might disrupt the excitatory synaptic transmission that promotes tumor growth [28, 29, 30]. Neurotrophic factor inhibitors like BDNF and other neurotrophic factors are implicated in tumor growth, inhibiting their signaling pathways (e.g., Trk inhibitors) could be explored for their anti‐tumor effects [29, 154]. Neuronal activity modulators drugs that alter neuronal activity, such as sodium channel blockers and GABA analogs used in epilepsy, might affect the hyperexcitability of neurons that contributes to tumor progression [61, 210, 211, 212, 213, 214, 215]. Synaptic plasticity modulators affect synaptic plasticity, such as those influencing long‐term potentiation (LTP) and long‐term depression (LTD), which could potentially alter the adaptive changes in the neural circuitry that support tumor growth [216, 217, 218, 219, 220, 221]. Paracrine signaling inhibitors, targeting paracrine factors like NLGN3, IGF‐1, and their downstream signaling pathways could disrupt the supportive TME for tumor cells [29, 45, 46].

Current practices involve the suppression of proteins and other substances secreted by neurons which can enhance tumor activity. For instance, NLGN3, a synaptic adhesion molecule, is targeted using its inhibitor Neurexin‐1b (NRXN1b), which binds specifically to NLGN3, depleting it [27]. ADAM10 inhibitors, such as INCB7839 and GI254023X, also intervene in the NLGN3 production pathway (NCT04295759). In addition, the NLGN3 knockout mice can also achieve tumor suppression effects in combination with the aforementioned treatments [31]. Given that TSP‐1 can serve as a regulatory factor for neuronal activity to drive glioma growth, researchers introduced gabapentin, a TSP‐1 receptor blocker, into the co‐culture medium of neurons and glioma cells. After 24–48 h of exposure, both the nerve pulses and the proliferation of HFC glioma cells co‐cultured with neurons were significantly decreased. Genetic shRNA targeting of TSP‐1 or pharmacological inhibition can similarly reduce GBM cell proliferation in the TME, laying the foundation for the development of therapeutic strategies that can improve cognition and survival [61]. Furthermore, genetic or pharmacological blockade of TrkB has been shown to impede the growth of pediatric gliomas, as evidenced by increased survival rates in mice with brainstem NTRK2‐KO invasive pediatric glioblastoma or frontal cortex NTRK2‐KO pediatric cortical glioblastoma, compared to controls to wild‐type controls [44].

It is crucial to acknowledge that while inhibiting these molecules can yield tumor‐suppressive effects in laboratory settings and mice, the broader implications of such interventions on brain function have not been fully explored. Researchers should consider the potential impact of inhibiting or knocking out proteins and synaptic structures on cognitive functions, and further studies involving animal models should assess effects on behavior, including cognitive and anxiety‐related responses. For example, the role of NLGN3 in the maturation of excitatory synapses and its broader impact on brain function during tumor intervention needs further investigation [129, 131, 222]. Additionally, BDNF's role in neuronal survival, synaptic plasticity, and neurogenesis, particularly its influence on LTP, which underpins learning and memory processes, must be evaluated to understand the full scope of potential cognitive effects on humans [44, 100, 223, 224, 225].

A complementary strategy involves disrupting the glioma network. Since traditional chemotherapy and radiation may not always be effective as aforementioned [226, 227, 228, 229], addressing glioma network resistance and the potential for calcium waves to worsen biological effects on tumor cells is vital [226, 227, 228, 229]. The molecular underpinnings that drive the formation, progression, and maintenance of TMs and their GJs present viable targets for pharmaceutical innovation. Upstream regulatory factors, such as GAP43 and TTYH1, are integral to the genesis and functionality of TMs and could emerge as promising candidates for therapeutic intervention [124, 125, 230, 231, 232, 233, 234, 235]. Currently, several clinical trials targeting the glioma network are in progress. For instance, in the treatment of recurrent adult glioblastoma, a combination therapy involving temozolomide chemotherapy is being tested to disrupt the glioma tumor network (MecMet/NOA‐24; EudraCT 2021‐000708‐39). Additionally, pirenzepine, an antiepileptic drug functioning as a non‐competitive AMPA receptor inhibitor, is being investigated for its potential to target glutamatergic neuronal‐glial synapses, thereby inhibiting the glioma network at the excitatory synapse level (EudraCT 2023–503,938‐52).

Given the crucial role of various secreted proteins and synaptic structures in the brain, it is our conviction that in treating intracranial tumors, interventions should be carefully targeted at the points of interaction between neurons and tumor cells, with a paramount focus on preserving the integrity of the surrounding neural tissue. Employing frameless stereotactic technology or image‐guided neurosurgery allows for the precise delivery of therapeutic agents to tumor tissues, thereby minimizing collateral damage to other brain regions [230, 236, 237, 238, 239, 240, 241, 242]. Concurrently, the administration of neuroprotective agents, which do not contribute to tumor progression, is imperative for safeguarding healthy brain tissues [243, 244]. Protecting neurons from damage, such as oxidative stress or inflammation, might also protect against the neurotoxic effects of tumors and support the health of the CNS microenvironment [245, 246, 247, 248, 249, 250]. With the advancement of fundamental research and the ongoing refinement of clinical trial methodologies, it is hopeful that we will soon develop efficacious treatments for intracranial tumors that spare brain function.

### 5.2 A New Perspective on Brain Tumors

Brain tumors are not isolated entities but are interconnected with neurons, glial cells, and proteins in their environment. The role of neurons provides an expanded perspective for investigating the mechanisms behind brain tumor emergence and progression.

Initially, innovative interactions among tumor cells, neurons, and other tumor cells have been discovered. The interaction between neurons and tumors has not been extensively studied, and the communication among tumor cells seems to have been similarly overlooked in previous research. However, the communication among immune cells within the TME is likely to be more frequent and intimate, involving a complex array of chemokine receptor signals and interleukins [251, 252, 253, 254, 255]. The field of cancer neuroscience, which explores the relationship between tumors and neurons, provides us with fresh insights into the microtubules and networks of brain tumors. Some cells within GBM are interconnected through TMs, forming a network that may be responsible for the resistance of tumors to various treatment modalities [84]. The formation of synapses between neurons and tumor cells may exploit these tumor networks to facilitate the proliferation and invasion of GBM [28, 84]. Notably, the intriguing observation that neurons and tumor cells can form synaptic structures indeed opens up a plethora of questions regarding the nature of these interactions and the functional capabilities of tumor cells within such an arrangement. In classical synapses, the postsynaptic density is a complex structure enriched with various proteins that are crucial for synaptic function, including receptors, scaffolding proteins, and enzymes [256, 257, 258, 259]. It would be valuable to investigate whether tumor cells forming synapses also develop a similar postsynaptic density (PSD), which is very common in classic synapses [260, 261, 262]. Identifying the specific proteins present and their organization could provide insights into the functional state of these atypical synapses. In addition, whether tumor cells undergoing synaptic integration will exhibit morphological changes similar to neurons is something we need to focus on in future research. Investigating these changes under microscopic examination could offer clues about the structural adaptations that enable synaptic communication. Furthermore, it would be essential to examine their electrophysiological properties to determine if tumor cells can truly function as postsynaptic elements, which have already been reported [28, 29, 30, 31, 85]. This includes assessing whether they can generate postsynaptic potentials (PSPs) in response to neurotransmitter release and whether they exhibit changes in membrane potential or ion channel activity. Finally, it is also important to consider whether these tumor‐associated synapses exhibit plasticity, similar to that seen in the nervous system, potentially manifesting as alterations in synaptic strength or structure in response to activity [28, 44, 84, 124].

Secondly, from the perspective of cancer neuroscience, it is imperative to re‐evaluate the underlying mechanisms that drive tumor metastasis. For example, breast cancer is the most common malignant tumor among women, with its metastasis mechanism being a multifaceted process [263, 264]. Historically, researchers have delved into a plethora of molecular mechanisms. Breast cancer metastasis typically unfolds through a series of sequential steps: local invasion by tumor cells, entry into the bloodstream or lymphatic system to become circulating tumor cells (CTCs), escape from circulation and colonization in distant tissues as disseminated tumor cells (DTCs), and ultimately transformation into metastasis‐initiating cells (MICs) that establish metastatic foci [35, 265, 266, 267]. Recent studies have uncovered novel molecular mechanisms linked to bone metastasis in breast cancer, such as ULK1 protein, FAM20C kinase, CENPF protein, and CTGF secreted by tumor cells [268, 269, 270, 271, 272, 273, 274, 275, 276]. These factors significantly contribute to the bone metastasis of breast cancer. Breast cancer metastasis exhibits a distinct organ preference, particularly for bone metastasis, which predominantly occurs in estrogen receptor (ER)‐positive luminal breast cancer (LBC) [277, 278]. It has been discovered that SCUBE2, secreted by tumors, is a pivotal factor in mediating the bone metastasis propensity in LBC [278]. The intriguing phenomenon previously mentioned, where tumors that metastasize to the brain from peripheral sites tend to express proteins typically found in the nervous system, appears to be relevant in these contexts as well. For instance, the FAM20C enzymes, implicated in the bone metastasis of breast cancer, are not commonly expressed in breast cells but are significant in bone tissue [274, 279]. Notably, as a secreted kinase, it is essential for osteoblast differentiation and function during bone development and the mineralization process [279, 280, 281]. The elevated expression of specific or key proteins within metastatic tissue before the onset of metastasis merits further exploration. The unique tripartite synaptic structure and its interaction with neurons have been elucidated for the first time. Based on electrophysiological studies and interventions on synapses and neurotransmitters, this structure may play a crucial role in inhibiting brain metastasis of breast cancer or directly suppressing breast cancer in the brain. However, there is a regrettable gap in our understanding of how this tripartite synapse is formed and the specific mechanisms of structural modification that promote brain metastasis of breast cancer, which warrants further exploration.

Thirdly, we have proposed new insights into the occurrence of tumors. Previous research has firmly established that neural precursor cells (NPCs) serve as a significant source of tumor cells, with their transformation into malignancy being a key pathway for the development of intracranial tumors [45, 46, 282, 283, 284, 285]. The conversion of NPCs into tumor cells is a multifaceted process that entails intricate molecular and cellular interactions. Genetically, mutations in crucial tumor suppressor genes—including TP53, NF1, and PTEN—and the activation of oncogenes like EGFR and PDGFRA can initiate cell proliferation, impede apoptosis, and foster tumorigenesis [185, 286, 287, 288, 289, 290, 291, 292, 293, 294].

Additionally, the tumor microenvironment's influence, characterized by the presence of oncogenic factors, the disruption of cellular signaling cascades, and alterations in cellular components—encompassing immune cells, endothelial cells, and astrocytes—as well as changes in the extracellular matrix (ECM), can collectively facilitate the malignant transformation of NPCs [286, 295, 296, 297, 298, 299, 300, 301, 302, 303, 304, 305, 306]. The role of neurons, as integral and central elements of the nervous system, in the initiation of cancer from precursor cells represents a novel and unanticipated dimension in our understanding of neuro‐oncogenesis. Within the framework of cancer neuroscience, research has illuminated that the secretion of BDNF and IGF‐1, coupled with direct synaptic interactions between glutamatergic and GABAergic neurons and OPCs, plays a crucial role in the malignant transformation of OPCs into glioma cells [43, 46, 79, 186, 187, 188, 189, 190]. However, there are numerous considerations that merit attention. For instance, while it is commonly believed that the sense of smell does not precipitate the development of glioma—a tumor originating from the neuroepithelium and potentially linked to factors such as ionizing radiation and viral infections—Liu's research presents compelling evidence that environmental stimuli can interact with our senses and potentially trigger cancer. In his study, gases were found to stimulate olfactory neurons, prompting other cells in the olfactory bulb to release IGF1, which in turn can stimulate the division of OPCs harboring pro‐cancer mutations [46, 307]. This suggests a significant interaction between our environment and senses that could lead to cancer. However, there is no evidence to suggest that analogous mechanisms are at play in humans. The mice used in Liu's experiments were genetically engineered to develop gliomas. If olfactory stimulation is to be considered a key factor in the genesis of human gliomas, a stronger genetic predisposition association may be necessary. Moreover, our comprehension of olfactory gliomas is in its infancy with this study. Currently, investigations into the carcinogenesis of neural precursor cells through neuronal intervention are primarily confined to OPCs. It is essential to expand research to encompass a broader spectrum of precursor cell types to gain a more comprehensive understanding of neuro‐oncogenesis.

Lastly, from the perspective of cancer neuroscience, we explore some differences between central primary tumors, such as gliomas, and brain metastases. The first point is that the origins and developments of them are different: primary CNS tumors, such as gliomas, meningiomas, and pituitary adenomas, arise from the cells native to the CNS and their neural regulation of their TME is influenced by the direct interactions between resident neurons and glial cells, which can contribute to tumor growth through paracrine signaling and the formation of synaptic connections [5, 36, 56, 308, 309, 310, 311, 312, 313, 314, 315, 316, 317, 318]. In contrast, metastatic tumors, such as those from lung, melanoma, and breast cancers, have already undergone the process of invasion and intravasation in their primary sites, and once in the CNS, these cells may adapt to the neural environment by altering their gene expression and protein production profiles to engage with neurons and other CNS cells [30, 113, 319, 320]. The second point is that the neural‐tumor interaction mechanisms differ markedly: primary CNS tumors can form direct synaptic connections with neurons, influencing their behavior through neurotransmitters and electrical signaling [24, 27, 28, 29, 45, 128]. Metastatic cells within the CNS may engage in unique synaptic interactions not typically seen with primary tumors, such as “pseudo tripartite synapses” between breast cancer cells and neurons, which can release glutamate and promote tumor growth and metastasis within the brain [30]. While both primary and metastatic tumors within the CNS are subject to neural regulation, the specific mechanisms and implications of these interactions can vary greatly. Primary tumors may have a more direct and continuous interaction with the CNS microenvironment, whereas metastatic tumors must adapt to a new environment that is distinct from their origin, potentially involving unique adaptations and signaling pathways. Gaining insight into these differences is crucial for developing targeted therapies that can effectively combat both types of CNS tumors.

## 6 Conclusions

Neurons significantly influence the development and growth of both primary and metastatic brain tumors. They are capable of establishing functional connections with tumor cells via chemical or electrical synapses, thereby directly or indirectly modulating the behavior of the tumor through intercellular coupling networks. Furthermore, neurons contribute to the TME by secreting proteins that can foster tumor growth and facilitate the establishment of synaptic connections with tumor cells. Neurons also transform OPCs into cancerous cells through synaptic and paracrine signaling. Targeting and interrupting these neuron‐to‐tumor pathways could potentially lead to the amelioration or halting of tumor advancement. Emerging therapeutic strategies are likely to concentrate on the development of pharmaceuticals designed to inhibit or neutralize the proteins and bioactive substances released by neurons. These drugs aim to sever synaptic links and interactions between infiltrating cells and tumor cells, as well as to disrupt the electrical coupling within glioma cell networks. Nonetheless, it is imperative to confront the unique challenges associated with the central nervous system, including the blood–brain barrier's impermeability to many drugs and the complexities inherent in brain tumor treatment, alongside the potential side effects of neuroactive medications. We hold a strong conviction that, with ongoing advancements in foundational research and the refinement of clinical trial methodologies, it is within our reach to devise more potent treatment approaches for intracranial tumors that concurrently safeguard vital brain functions.

## Author Contributions

W.L. wrote the main part of the article. Y.W. guided writing, offering suggestions to revise, and reviewed and agreed upon the final version of the manuscript. All authors read and approved the final manuscript.

## Ethics Statement

The authors have nothing to report.

## Consent

The authors have nothing to report.

## Conflicts of Interest

The authors declare no conflicts of interest.

## Data Availability

The authors have nothing to report.

## References

[1] R. J. Wilson , C. D. Thomas , R. Fox , D. B. Roy , and W. E. Kunin , “Spatial Patterns in Species Distributions Reveal Biodiversity Change,” Nature 432, no. 7015 (2004): 393–396. 15549106 10.1038/nature03031

[2] S. C. Mack , C. G. Hubert , T. E. Miller , M. D. Taylor , and J. N. Rich , “An Epigenetic Gateway to Brain Tumor Cell Identity,” Nature Neuroscience 19, no. 1 (2015): 10–19. 10.1038/nn.4190 PMC5568053 26713744

[3] J. H. Sampson , M. V. Maus , and C. H. June , “Immunotherapy for Brain Tumors,” Journal of Clinical Oncology 35, no. 21 (2017): 2450–2456. 28640704 10.1200/JCO.2017.72.8089

[4] A. Boire , P. K. Brastianos , L. Garzia , and M. Valiente , “Brain Metastasis,” Nature Reviews Cancer 20, no. 1 (2019): 4–11. 31780784 10.1038/s41568-019-0220-y

[5] M. J. van den Bent , M. Geurts , P. J. French , et al., “Primary Brain Tumours in Adults,” Lancet 402, no. 10412 (2023): 1564–1579. 37738997 10.1016/S0140-6736(23)01054-1

[6] Q. T. Ostrom , M. Price , C. Neff , et al., “CBTRUS Statistical Report: Primary Brain and Other Central Nervous System Tumors Diagnosed in the United States in 2015–2019,” Neuro‐Oncology 24, no. Supplement_5 (2022): v1–v95. 36196752 10.1093/neuonc/noac202 PMC9533228

[7] A. P. Patel , J. L. Fisher , E. Nichols , et al., “Global, Regional, and National Burden of Brain and Other CNS Cancer, 1990–2016: A Systematic Analysis for the Global Burden of Disease Study 2016,” Lancet Neurology 18, no. 4 (2019): 376–393. 30797715 10.1016/S1474-4422(18)30468-X PMC6416167

[8] M. Zaidi , “Skeletal Remodeling in Health and Disease,” Nature Medicine 13, no. 7 (2007): 791–801. 10.1038/nm1593 17618270

[9] C. L. Sisk and D. L. Foster , “The Neural Basis of Puberty and Adolescence,” Nature Neuroscience 7, no. 10 (2004): 1040–1047. 15452575 10.1038/nn1326

[10] K. L. Gamble , R. Berry , S. J. Frank , and M. E. Young , “Circadian Clock Control of Endocrine Factors,” Nature Reviews Endocrinology 10, no. 8 (2014): 466–475. 10.1038/nrendo.2014.78 PMC4304769 24863387

[11] M. Schiller , T. L. Ben‐Shaanan , and A. Rolls , “Neuronal Regulation of Immunity: Why, How and Where?,” Nature Reviews Immunology 21, no. 1 (2020): 20–36. 10.1038/s41577-020-0387-1 32811994

[12] C. Gizowski and C. W. Bourque , “The Neural Basis of Homeostatic and Anticipatory Thirst,” Nature Reviews Nephrology 14, no. 1 (2017): 11–25. 29129925 10.1038/nrneph.2017.149

[13] B. B. Lowell , “New Neuroscience of Homeostasis and Drives for Food, Water, and Salt,” New England Journal of Medicine 380, no. 5 (2019): 459–471. 30699320 10.1056/NEJMra1812053

[14] C. Perez‐Castro , U. Renner , M. R. Haedo , G. K. Stalla , and E. Arzt , “Cellular and Molecular Specificity of Pituitary Gland Physiology,” Physiological Reviews 92, no. 1 (2012): 1–38. 22298650 10.1152/physrev.00003.2011

[15] T. Liu , Y. Xu , C. X. Yi , Q. Tong , and D. Cai , “The Hypothalamus for Whole‐Body Physiology: From Metabolism to Aging,” Protein and Cell 13, no. 6 (2021): 394–421. 33826123 10.1007/s13238-021-00834-x PMC9095790

[16] U. Albrecht and J. A. Ripperger , “Circadian Clocks and Sleep: Impact of Rhythmic Metabolism and Waste Clearance on the Brain,” Trends in Neurosciences 41, no. 10 (2018): 677–688. 30274603 10.1016/j.tins.2018.07.007

[17] O. Avraham , P. Y. Deng , S. Jones , et al., “Satellite Glial Cells Promote Regenerative Growth in Sensory Neurons,” Nature Communications 11, no. 1 (2020): 4891. 10.1038/s41467-020-18642-y PMC7524726 32994417

[18] P. Kofuji and A. Araque , “Astrocytes and Behavior,” Annual Review of Neuroscience 44, no. 1 (2021): 49–67. 10.1146/annurev-neuro-101920-112225 PMC8257756 33406370

[19] K.‐A. Nave and H. B. Werner , “Ensheathment and Myelination of Axons: Evolution of Glial Functions,” Annual Review of Neuroscience 44, no. 1 (2021): 197–219. 10.1146/annurev-neuro-100120-122621 33722070

[20] M. Yazdankhah , P. Shang , S. Ghosh , et al., “Role of Glia in Optic Nerve,” Progress in Retinal and Eye Research 81 (2021): 100886. 32771538 10.1016/j.preteyeres.2020.100886 PMC7865017

[21] D. D. Liu , J. Q. He , R. Sinha , et al., “Purification and Characterization of Human Neural Stem and Progenitor Cells,” Cell 186, no. 6 (2023): 1179–1194.e15. 36931245 10.1016/j.cell.2023.02.017 PMC10409303

[22] K. R. Taylor and M. Monje , “Neuron–Oligodendroglial Interactions in Health and Malignant Disease,” Nature Reviews Neuroscience 24, no. 12 (2023): 733–746. 37857838 10.1038/s41583-023-00744-3 PMC10859969

[23] M. Monje , J. C. Borniger , N. J. D'Silva , et al., “Roadmap for the Emerging Field of Cancer Neuroscience,” Cell 181, no. 2 (2020): 219–222. 32302564 10.1016/j.cell.2020.03.034 PMC7286095

[24] F. Winkler , H. S. Venkatesh , M. Amit , et al., “Cancer Neuroscience: State of the Field, Emerging Directions,” Cell 186, no. 8 (2023): 1689–1707. 37059069 10.1016/j.cell.2023.02.002 PMC10107403

[25] D. D. Shi , J. A. Guo , H. I. Hoffman , et al., “Therapeutic Avenues for Cancer Neuroscience: Translational Frontiers and Clinical Opportunities,” Lancet Oncology 23, no. 2 (2022): e62–e74. 35114133 10.1016/S1470-2045(21)00596-9 PMC9516432

[26] I. E. Demir , C. Mota Reyes , W. Alrawashdeh , et al., “Future Directions in Preclinical and Translational Cancer Neuroscience Research,” Nature Cancer 1, no. 11 (2020): 1027–1031. 34327335 10.1038/s43018-020-00146-9 PMC8315010

[27] H. S. Venkatesh , T. B. Johung , V. Caretti , et al., “Neuronal Activity Promotes Glioma Growth Through Neuroligin‐3 Secretion,” Cell 161, no. 4 (2015): 803–816. 25913192 10.1016/j.cell.2015.04.012 PMC4447122

[28] V. Venkataramani , D. I. Tanev , C. Strahle , et al., “Glutamatergic Synaptic Input to Glioma Cells Drives Brain Tumour Progression,” Nature 573, no. 7775 (2019): 532–538. 31534219 10.1038/s41586-019-1564-x

[29] H. S. Venkatesh , W. Morishita , A. C. Geraghty , et al., “Electrical and Synaptic Integration of Glioma Into Neural Circuits,” Nature 573, no. 7775 (2019): 539–545. 31534222 10.1038/s41586-019-1563-y PMC7038898

[30] Q. Zeng , I. P. Michael , P. Zhang , et al., “Synaptic Proximity Enables NMDAR Signalling to Promote Brain Metastasis,” Nature 573, no. 7775 (2019): 526–531. 31534217 10.1038/s41586-019-1576-6 PMC6837873

[31] M. Monje , “Synaptic Communication in Brain Cancer,” Cancer Research 80, no. 14 (2020): 2979–2982. 32381657 10.1158/0008-5472.CAN-20-0646 PMC7367763

[32] R. Mancusi and M. Monje , “The Neuroscience of Cancer,” Nature 618, no. 7965 (2023): 467–479. 37316719 10.1038/s41586-023-05968-y PMC11146751

[33] E. Jung , J. Alfonso , M. Osswald , H. Monyer , W. Wick , and F. Winkler , “Emerging Intersections Between Neuroscience and Glioma Biology,” Nature Neuroscience 22, no. 12 (2019): 1951–1960. 31719671 10.1038/s41593-019-0540-y

[34] M. L. Broekman , S. L. N. Maas , E. R. Abels , T. R. Mempel , A. M. Krichevsky , and X. O. Breakefield , “Multidimensional Communication in the Microenvirons of Glioblastoma,” Nature Reviews Neurology 14, no. 8 (2018): 482–495. 29985475 10.1038/s41582-018-0025-8 PMC6425928

[35] A. S. Achrol , R. C. Rennert , C. Anders , et al., “Brain metastases,” Nature Reviews Disease Primers 5 (2019): 5. 10.1038/s41572-018-0055-y 30655533

[36] F. Klemm , R. R. Maas , R. L. Bowman , et al., “Interrogation of the Microenvironmental Landscape in Brain Tumors Reveals Disease‐Specific Alterations of Immune Cells,” Cell 181, no. 7 (2020): 1643–1660.e17. 32470396 10.1016/j.cell.2020.05.007 PMC8558904

[37] A. Bikfalvi , C. A. da Costa , T. Avril , et al., “Challenges in Glioblastoma Research: Focus on the Tumor Microenvironment,” Trends in Cancer 9, no. 1 (2023): 9–27. 36400694 10.1016/j.trecan.2022.09.005

[38] D. Hanahan and M. Monje , “Cancer Hallmarks Intersect With Neuroscience in the Tumor Microenvironment,” Cancer Cell 41, no. 3 (2023): 573–580. 36917953 10.1016/j.ccell.2023.02.012 PMC10202656

[39] Y. Hoogstrate , K. Draaisma , S. A. Ghisai , et al., “Transcriptome Analysis Reveals Tumor Microenvironment Changes in Glioblastoma,” Cancer Cell 41, no. 4 (2023): 678–692 e7. 36898379 10.1016/j.ccell.2023.02.019

[40] N. Khanmammadova , S. Islam , P. Sharma , and M. Amit , “Neuro‐Immune Interactions and Immuno‐Oncology,” Trends Cancer 9, no. 8 (2023): 636–649. 37258398 10.1016/j.trecan.2023.05.002 PMC10524972

[41] L. Bejarano , A. Kauzlaric , E. Lamprou , et al., “Interrogation of Endothelial and Mural Cells in Brain Metastasis Reveals Key Immune‐Regulatory Mechanisms,” Cancer Cell 42, no. 3 (2024): 378–395 e10. 38242126 10.1016/j.ccell.2023.12.018

[42] A. C. Greenwald , N. G. Darnell , R. Hoefflin , et al., “Integrative Spatial Analysis Reveals a Multi‐Layered Organization of Glioblastoma,” Cell 187, no. 10 (2024): 2485–2501 e26. 38653236 10.1016/j.cell.2024.03.029 PMC11088502

[43] Y. Pan , J. D. Hysinger , T. Barron , et al., “NF1 Mutation Drives Neuronal Activity‐Dependent Initiation of Optic Glioma,” Nature 594, no. 7862 (2021): 277–282. 34040258 10.1038/s41586-021-03580-6 PMC8346229

[44] K. R. Taylor , T. Barron , A. Hui , et al., “Glioma Synapses Recruit Mechanisms of Adaptive Plasticity,” Nature 623, no. 7986 (2023): 366–374. 37914930 10.1038/s41586-023-06678-1 PMC10632140

[45] H. S. Venkatesh , L. T. Tam , P. J. Woo , et al., “Targeting Neuronal Activity‐Regulated Neuroligin‐3 Dependency in High‐Grade Glioma,” Nature 549, no. 7673 (2017): 533–537. 28959975 10.1038/nature24014 PMC5891832

[46] P. Chen , W. Wang , R. Liu , et al., “Olfactory Sensory Experience Regulates Gliomagenesis via Neuronal IGF1,” Nature 606, no. 7914 (2022): 550–556. 35545672 10.1038/s41586-022-04719-9

[47] D. Hambardzumyan , D. H. Gutmann , and H. Kettenmann , “The Role of Microglia and Macrophages in Glioma Maintenance and Progression,” Nature Neuroscience 19, no. 1 (2016): 20–27. 26713745 10.1038/nn.4185 PMC4876023

[48] W. Li and M. B. Graeber , “The Molecular Profile of Microglia Under the Influence of Glioma,” Neuro‐Oncology 14, no. 8 (2012): 958–978. 22573310 10.1093/neuonc/nos116 PMC3408253

[49] T. Li , D. Xu , Z. Ruan , et al., “Metabolism/Immunity Dual‐Regulation Thermogels Potentiating Immunotherapy of Glioblastoma Through Lactate‐Excretion Inhibition and PD‐1/PD‐L1 Blockade,” Advanced science 11, no. 18 (2024): e2310163. 38460167 10.1002/advs.202310163 PMC11095231

[50] M. A. Jayaram and J. J. Phillips , “Role of the Microenvironment in Glioma Pathogenesis,” Annual Review of Pathology: Mechanisms of Disease 19, no. 1 (2024): 181–201. 10.1146/annurev-pathmechdis-051122-110348 37832944

[51] V. M. Ravi , P. Will , J. Kueckelhaus , et al., “Spatially Resolved Multi‐Omics Deciphers Bidirectional Tumor‐Host Interdependence in Glioblastoma,” Cancer Cell 40, no. 6 (2022): 639–655.e13. 35700707 10.1016/j.ccell.2022.05.009

[52] Q. Wang , B. Hu , X. Hu , et al., “Tumor Evolution of Glioma‐Intrinsic Gene Expression Subtypes Associates With Immunological Changes in the Microenvironment,” Cancer Cell 32, no. 1 (2017): 42–56 e6. 28697342 10.1016/j.ccell.2017.06.003 PMC5599156

[53] M. Yao , P. B. Ventura , Y. Jiang , et al., “Astrocytic Trans‐Differentiation Completes a Multicellular Paracrine Feedback Loop Required for Medulloblastoma Tumor Growth,” Cell 180, no. 3 (2020): 502–520.e19. 31983537 10.1016/j.cell.2019.12.024 PMC7259679

[54] A. de Pablos‐Aragoneses and M. Valiente , “An Inbred Ecosystem That Supports Medulloblastoma,” Immunity 52, no. 3 (2020): 431–433. 32187514 10.1016/j.immuni.2020.02.010

[55] L. van Hooren , S. M. Handgraaf , D. J. Kloosterman , et al., “CD103(+) Regulatory T Cells Underlie Resistance to Radio‐Immunotherapy and Impair CD8(+) T Cell Activation in Glioblastoma,” Nature Cancer 4, no. 5 (2023): 665–681. 37081259 10.1038/s43018-023-00547-6 PMC10212765

[56] B. M. Andersen , C. Faust Akl , M. A. Wheeler , E. A. Chiocca , D. A. Reardon , and F. J. Quintana , “Glial and Myeloid Heterogeneity in the Brain Tumour Microenvironment,” Nature Reviews Cancer 21, no. 12 (2021): 786–802. 34584243 10.1038/s41568-021-00397-3 PMC8616823

[57] C. Zhu , J. M. Kros , C. Cheng , and D. Mustafa , “The Contribution of Tumor‐Associated Macrophages in Glioma Neo‐Angiogenesis and Implications for Anti‐Angiogenic Strategies,” Neuro‐Oncology 19, no. 11 (2017): 1435–1446. 28575312 10.1093/neuonc/nox081 PMC5737221

[58] X. Wang , H. Ding , Z. Li , et al., “Exploration and Functionalization of M1‐Macrophage Extracellular Vesicles for Effective Accumulation in Glioblastoma and Strong Synergistic Therapeutic Effects,” Signal Transduction and Targeted Therapy 7, no. 1 (2022): 74. 35292619 10.1038/s41392-022-00894-3 PMC8924195

[59] S. C. Buckingham , S. L. Campbell , B. R. Haas , et al., “Glutamate Release by Primary Brain Tumors Induces Epileptic Activity,” Nature Medicine 17, no. 10 (2011): 1269–1274. 10.1038/nm.2453 PMC3192231 21909104

[60] A. Meyer‐Baese , K. Jütten , U. Meyer‐Baese , et al., “Controllability and Robustness of Functional and Structural Connectomic Networks in Glioma Patients,” Cancers 15, no. 10 (2023): 2714. 37345051 10.3390/cancers15102714 PMC10216571

[61] S. Krishna , A. Choudhury , M. B. Keough , et al., “Glioblastoma Remodelling of Human Neural Circuits Decreases Survival,” Nature 617, no. 7961 (2023): 599–607. 37138086 10.1038/s41586-023-06036-1 PMC10191851

[62] A. A. Aabedi , B. Lipkin , J. Kaur , et al., “Functional Alterations in Cortical Processing of Speech in Glioma‐Infiltrated Cortex,” Proceedings of the National Academy of Sciences of the United States of America 118, no. 46 (2021): e2108959118. 34753819 10.1073/pnas.2108959118 PMC8609626

[63] N. M. Teplyuk , E. J. Uhlmann , A. H. K. Wong , et al., “MicroRNA‐10b Inhibition Reduces E2F1‐Mediated Transcription and miR‐15/16 Activity in Glioblastoma,” Oncotarget 6, no. 6 (2015): 3770–3783. 25738367 10.18632/oncotarget.3009 PMC4414152

[64] K. Herrup and Y. Yang , “Cell Cycle Regulation in the Postmitotic Neuron: Oxymoron or New Biology?,” Nature Reviews. Neuroscience 8, no. 5 (2007): 368–378. 17453017 10.1038/nrn2124

[65] Y. Zhu , Y. Lu , Q. Zhang , et al., “MicroRNA‐26a/b and Their Host Genes Cooperate to Inhibit the G1/S Transition by Activating the pRb Protein,” Nucleic Acids Research 40, no. 10 (2012): 4615–4625. 22210897 10.1093/nar/gkr1278 PMC3378857

[66] H. Kim , W. Huang , X. Jiang , B. Pennicooke , P. J. Park , and M. D. Johnson , “Integrative Genome Analysis Reveals an Oncomir/Oncogene Cluster Regulating Glioblastoma Survivorship,” Proceedings of the National Academy of Sciences of the United States of America 107, no. 5 (2010): 2183–2188. 20080666 10.1073/pnas.0909896107 PMC2836668

[67] M. F. Johnston , S. A. Simon , and F. Ramón , “Interaction of Anaesthetics With Electrical Synapses,” Nature 286, no. 5772 (1980): 498–500. 6250068 10.1038/286498a0

[68] T. C. Südhof , “Neuroligins and Neurexins Link Synaptic Function to Cognitive Disease,” Nature 455, no. 7215 (2008): 903–911. 18923512 10.1038/nature07456 PMC2673233

[69] M. T. Harnett , J. K. Makara , N. Spruston , W. L. Kath , and J. C. Magee , “Synaptic Amplification by Dendritic Spines Enhances Input Cooperativity,” Nature 491, no. 7425 (2012): 599–602. 23103868 10.1038/nature11554 PMC3504647

[70] S. Trenholm , A. J. McLaughlin , D. J. Schwab , et al., “Nonlinear Dendritic Integration of Electrical and Chemical Synaptic Inputs Drives Fine‐Scale Correlations,” Nature Neuroscience 17, no. 12 (2014): 1759–1766. 25344631 10.1038/nn.3851 PMC4265022

[71] G. Perea , M. Navarrete , and A. Araque , “Tripartite Synapses: Astrocytes Process and Control Synaptic Information,” Trends in Neurosciences 32, no. 8 (2009): 421–431. 19615761 10.1016/j.tins.2009.05.001

[72] A. C. Dolphin and A. Lee , “Presynaptic Calcium Channels: Specialized Control of Synaptic Neurotransmitter Release,” Nature Reviews Neuroscience 21, no. 4 (2020): 213–229. 32161339 10.1038/s41583-020-0278-2 PMC7873717

[73] A. E. Pereda , “Electrical Synapses and Their Functional Interactions With Chemical Synapses,” Nature Reviews Neuroscience 15, no. 4 (2014): 250–263. 24619342 10.1038/nrn3708 PMC4091911

[74] N. X. Tritsch , A. J. Granger , and B. L. Sabatini , “Mechanisms and Functions of GABA Co‐Release,” Nature Reviews Neuroscience 17, no. 3 (2016): 139–145. 26865019 10.1038/nrn.2015.21 PMC6980171

[75] J. Nagai , A. K. Rajbhandari , M. R. Gangwani , et al., “Hyperactivity With Disrupted Attention by Activation of an Astrocyte Synaptogenic Cue,” Cell 177, no. 5 (2019): 1280–1292.e20. 31031006 10.1016/j.cell.2019.03.019 PMC6526045

[76] S. Ghorbani and V. W. Yong , “The Extracellular Matrix as Modifier of Neuroinflammation and Remyelination in Multiple Sclerosis,” Brain 144, no. 7 (2021): 1958–1973. 33889940 10.1093/brain/awab059 PMC8370400

[77] M. Kaneko and M. P. Stryker , “Production of Brain‐Derived Neurotrophic Factor Gates Plasticity in Developing Visual Cortex,” Proceedings of the National Academy of Sciences of the United States of America 120 (2023): e2214833120. 36634145 10.1073/pnas.2214833120 PMC9934058

[78] Z. Pei , X. Guo , F. Zheng , et al., “Xuefu Zhuyu Decoction Promotes Synaptic Plasticity by Targeting miR‐191a‐5p/BDNF‐TrkB Axis in Severe Traumatic Brain Injury,” Phytomedicine 129 (2024): 155566. 38565001 10.1016/j.phymed.2024.155566

[79] S.‐c. Lin and D. E. Bergles , “Synaptic Signaling Between GABAergic Interneurons and Oligodendrocyte Precursor Cells in the Hippocampus,” Nature Neuroscience 7, no. 1 (2003): 24–32. 14661022 10.1038/nn1162

[80] R. A. Nicoll , S. Tomita , and D. S. Bredt , “Auxiliary Subunits Assist AMPA‐Type Glutamate Receptors,” Science 311, no. 5765 (2006): 1253–1256. 16513974 10.1126/science.1123339

[81] V. A. Derkach , M. C. Oh , E. S. Guire , and T. R. Soderling , “Regulatory Mechanisms of AMPA Receptors in Synaptic Plasticity,” Nature Reviews Neuroscience 8, no. 2 (2007): 101–113. 17237803 10.1038/nrn2055

[82] K. M. Harris and R. J. Weinberg , “Ultrastructure of Synapses in the Mammalian Brain,” Cold Spring Harbor Perspectives in Biology 4, no. 5 (2012): a005587. 22357909 10.1101/cshperspect.a005587 PMC3331701

[83] F. Winkler and W. Wick , “Harmful Networks in the Brain and Beyond,” Science 359, no. 6380 (2018): 1100–1101. 29590028 10.1126/science.aar5555

[84] M. Osswald , E. Jung , F. Sahm , et al., “Brain Tumour Cells Interconnect to a Functional and Resistant Network,” Nature 528, no. 7580 (2015): 93–98. 26536111 10.1038/nature16071

[85] R. Xie , T. Kessler , J. Grosch , et al., “Tumor Cell Network Integration in Glioma Represents a Stemness Feature,” Neuro‐Oncology 23, no. 5 (2021): 757–769. 33320195 10.1093/neuonc/noaa275 PMC8099480

[86] J. P. Dupuis , O. Nicole , and L. Groc , “NMDA Receptor Functions in Health and Disease: Old Actor, New Dimensions,” Neuron 111, no. 15 (2023): 2312–2328. 37236178 10.1016/j.neuron.2023.05.002

[87] L. V. Kalia , S. K. Kalia , and M. W. Salter , “NMDA Receptors in Clinical Neurology: Excitatory Times Ahead,” Lancet Neurology 7, no. 8 (2008): 742–755. 18635022 10.1016/S1474-4422(08)70165-0 PMC3589564

[88] L. Li , Q. Zeng , A. Bhutkar , et al., “GKAP Acts as a Genetic Modulator of NMDAR Signaling to Govern Invasive Tumor Growth,” Cancer Cell 33, no. 4 (2018): 736–751.e5. 29606348 10.1016/j.ccell.2018.02.011 PMC5896248

[89] L. M. Merlo , J. W. Pepper , B. J. Reid , and C. C. Maley , “Cancer as an Evolutionary and Ecological Process,” Nature Reviews. Cancer 6, no. 12 (2006): 924–935. 17109012 10.1038/nrc2013

[90] D. X. Nguyen , P. D. Bos , and J. Massague , “Metastasis: From Dissemination to Organ‐Specific Colonization,” Nature Reviews. Cancer 9, no. 4 (2009): 274–284. 19308067 10.1038/nrc2622

[91] Y. Liu and X. Cao , “Characteristics and Significance of the Pre‐Metastatic Niche,” Cancer Cell 30, no. 5 (2016): 668–681. 27846389 10.1016/j.ccell.2016.09.011

[92] J. Massague and A. C. Obenauf , “Metastatic Colonization by Circulating Tumour Cells,” Nature 529, no. 7586 (2016): 298–306. 26791720 10.1038/nature17038 PMC5029466

[93] L. Li and D. Hanahan , “Hijacking the Neuronal NMDAR Signaling Circuit to Promote Tumor Growth and Invasion,” Cell 153, no. 1 (2013): 86–100. 23540692 10.1016/j.cell.2013.02.051

[94] J. Yin , G. Tu , M. Peng , et al., “GPER‐Regulated lncRNA‐Glu Promotes Glutamate Secretion to Enhance Cellular Invasion and Metastasis in Triple‐Negative Breast Cancer,” FASEB Journal 34, no. 3 (2020): 4557–4572. 32030797 10.1096/fj.201901384RR

[95] K. U. Bayer , P. de Koninck , A. S. Leonard , J. W. Hell , and H. Schulman , “Interaction With the NMDA Receptor Locks CaMKII in an Active Conformation,” Nature 411, no. 6839 (2001): 801–805. 11459059 10.1038/35081080

[96] J. A. Matta , M. C. Ashby , A. Sanz‐Clemente , K. W. Roche , and J. T. R. Isaac , “mGluR5 and NMDA Receptors Drive the Experience‐ and Activity‐Dependent NMDA Receptor NR2B to NR2A Subunit Switch,” Neuron 70, no. 2 (2011): 339–351. 21521618 10.1016/j.neuron.2011.02.045 PMC3087383

[97] D. R. Grosshans , D. A. Clayton , S. J. Coultrap , and M. D. Browning , “LTP Leads to Rapid Surface Expression of NMDA but Not AMPA Receptors in Adult Rat CA1,” Nature Neuroscience 5, no. 1 (2002): 27–33. 11740502 10.1038/nn779

[98] A. Terashima , K. A. Pelkey , J. C. Rah , et al., “An Essential Role for PICK1 in NMDA Receptor‐Dependent Bidirectional Synaptic Plasticity,” Neuron 57, no. 6 (2008): 872–882. 18367088 10.1016/j.neuron.2008.01.028 PMC2336895

[99] S. J. Lee , Y. Escobedo‐Lozoya , E. M. Szatmari , and R. Yasuda , “Activation of CaMKII in Single Dendritic Spines During Long‐Term Potentiation,” Nature 458, no. 7236 (2009): 299–304. 19295602 10.1038/nature07842 PMC2719773

[100] S. C. Harward , N. G. Hedrick , C. E. Hall , et al., “Autocrine BDNF‐TrkB Signalling Within a Single Dendritic Spine,” Nature 538, no. 7623 (2016): 99–103. 27680698 10.1038/nature19766 PMC5398094

[101] H. J. Chen , M. Rojas‐Soto , A. Oguni , and M. B. Kennedy , “A Synaptic Ras‐GTPase Activating Protein (p135 SynGAP) Inhibited by CaM Kinase II,” Neuron 20, no. 5 (1998): 895–904. 9620694 10.1016/s0896-6273(00)80471-7

[102] H. C. Kornau , L. T. Schenker , M. B. Kennedy , and P. H. Seeburg , “Domain Interaction Between NMDA Receptor Subunits and the Postsynaptic Density Protein PSD‐95,” Science 269, no. 5231 (1995): 1737–1740. 7569905 10.1126/science.7569905

[103] S. Naisbitt , E. Kim , J. C. Tu , et al., “Shank, a Novel Family of Postsynaptic Density Proteins That Binds to the NMDA Receptor/PSD‐95/GKAP Complex and Cortactin,” Neuron 23, no. 3 (1999): 569–582. 10433268 10.1016/s0896-6273(00)80809-0

[104] G. Krapivinsky , I. Medina , L. Krapivinsky , S. Gapon , and D. E. Clapham , “SynGAP‐MUPP1‐CaMKII Synaptic Complexes Regulate p38 MAP Kinase Activity and NMDA Receptor‐Dependent Synaptic AMPA Receptor Potentiation,” Neuron 43, no. 4 (2004): 563–574. 15312654 10.1016/j.neuron.2004.08.003

[105] M. J. Schmeisser , E. Ey , S. Wegener , et al., “Autistic‐Like Behaviours and Hyperactivity in Mice Lacking ProSAP1/Shank2,” Nature 486, no. 7402 (2012): 256–260. 22699619 10.1038/nature11015

[106] Z. Ma , K. Liu , X. R. Li , et al., “Alpha‐Synuclein Is Involved in Manganese‐Induced Spatial Memory and Synaptic Plasticity Impairments via TrkB/Akt/Fyn‐Mediated Phosphorylation of NMDA Receptors,” Cell Death and Disease 11, no. 10 (2020): 834. 33033239 10.1038/s41419-020-03051-2 PMC7545185

[107] Q. Cai , M. Zeng , X. Wu , et al., “CaMKIIalpha‐Driven, Phosphatase‐Checked Postsynaptic Plasticity via Phase Separation,” Cell Research 31, no. 1 (2021): 37–51. 33235361 10.1038/s41422-020-00439-9 PMC7852677

[108] B. Compans , C. Camus , E. Kallergi , et al., “NMDAR‐Dependent Long‐Term Depression Is Associated With Increased Short Term Plasticity Through Autophagy Mediated Loss of PSD‐95,” Nature Communications 12, no. 1 (2021): 2849. 10.1038/s41467-021-23133-9 PMC8121912 33990590

[109] M. Yao , M. Meng , X. Yang , et al., “POSH Regulates Assembly of the NMDAR/PSD‐95/Shank Complex and Synaptic Function,” Cell Reports 39, no. 1 (2022): 110642. 35385725 10.1016/j.celrep.2022.110642

[110] Z. Shen , D. Sun , A. Savastano , et al., “Multivalent Tau/PSD‐95 Interactions Arrest In Vitro Condensates and Clusters Mimicking the Postsynaptic Density,” Nature Communications 14, no. 1 (2023): 6839. 10.1038/s41467-023-42295-2 PMC10611757 37891164

[111] E. S. Park , S. J. Kim , S. W. Kim , et al., “Cross‐Species Hybridization of Microarrays for Studying Tumor Transcriptome of Brain Metastasis,” Proceedings of the National Academy of Sciences of the United States of America 108, no. 42 (2011): 17456–17461. 21987811 10.1073/pnas.1114210108 PMC3198333

[112] J. Neman , J. Termini , S. Wilczynski , et al., “Human Breast Cancer Metastases to the Brain Display GABAergic Properties in the Neural Niche,” Proceedings of the National Academy of Sciences of the United States of America 111, no. 3 (2014): 984–989. 24395782 10.1073/pnas.1322098111 PMC3903266

[113] F. Qu , S. C. Brough , W. Michno , et al., “Crosstalk Between Small‐Cell Lung Cancer Cells and Astrocytes Mimics Brain Development to Promote Brain Metastasis,” Nature Cell Biology 25, no. 10 (2023): 1506–1519. 37783795 10.1038/s41556-023-01241-6 PMC11230587

[114] A. Vílchez‐Acosta , Y. Manso , A. Cárdenas , et al., “Specific Contribution of Reelin Expressed by Cajal–Retzius Cells or GABAergic Interneurons to Cortical Lamination,” Proceedings of the National Academy of Sciences of the United States of America 119 (2022): e2120079119. 36067316 10.1073/pnas.2120079119 PMC9477240

[115] J. A. Del Río , B. Heimrich , V. Borrell , et al., “A Role for Cajal‐Retzius Cells and Reelin in the Development of Hippocampal Connections,” Nature 385, no. 6611 (1997): 70–74. 8985248 10.1038/385070a0

[116] E. Huang‐Hobbs , Y. T. Cheng , Y. Ko , et al., “Remote Neuronal Activity Drives Glioma Progression Through SEMA4F,” Nature 619, no. 7971 (2023): 844–850. 37380778 10.1038/s41586-023-06267-2 PMC10840127

[117] G. Söhl , S. Maxeiner , and K. Willecke , “Expression and Functions of Neuronal Gap Junctions,” Nature Reviews Neuroscience 6, no. 3 (2005): 191–200. 15738956 10.1038/nrn1627

[118] P. Alcamí and A. E. Pereda , “Beyond Plasticity: The Dynamic Impact of Electrical Synapses on Neural Circuits,” Nature Reviews Neuroscience 20, no. 5 (2019): 253–271. 30824857 10.1038/s41583-019-0133-5

[119] C. Giaume , C. C. Naus , J. C. Sáez , and L. Leybaert , “Glial Connexins and Pannexins in the Healthy and Diseased Brain,” Physiological Reviews 101, no. 1 (2021): 93–145. 32326824 10.1152/physrev.00043.2018

[120] M. V. Sofroniew , “HepaCAM Shapes Astrocyte Territories, Stabilizes Gap‐Junction Coupling, and Influences Neuronal Excitability,” Neuron 109, no. 15 (2021): 2365–2367. 34352210 10.1016/j.neuron.2021.07.010

[121] A. Pedroni , Y. W. E. Dai , L. Lafouasse , et al., “Neuroprotective Gap‐Junction‐Mediated Bystander Transformations in the Adult Zebrafish Spinal Cord After Injury,” Nature Communications 15, no. 1 (2024): 4331. 10.1038/s41467-024-48729-9 PMC11109231 38773121

[122] C. Ni , X. Lou , X. Yao , et al., “ZIP1+ Fibroblasts Protect Lung Cancer Against Chemotherapy via Connexin‐43 Mediated Intercellular Zn2+ Transfer,” Nature Communications 13, no. 1 (2022): 5919. 10.1038/s41467-022-33521-4 PMC9547061 36207295

[123] D. Hausmann , D. C. Hoffmann , V. Venkataramani , et al., “Autonomous Rhythmic Activity in Glioma Networks Drives Brain Tumour Growth,” Nature 613, no. 7942 (2022): 179–186. 36517594 10.1038/s41586-022-05520-4

[124] E. Jung , M. Osswald , J. Blaes , et al., “Tweety‐Homolog 1 Drives Brain Colonization of Gliomas,” Journal of Neuroscience 37, no. 29 (2017): 6837–6850. 28607172 10.1523/JNEUROSCI.3532-16.2017 PMC6705725

[125] V. Venkataramani , M. Schneider , F. A. Giordano , et al., “Disconnecting Multicellular Networks in Brain Tumours,” Nature Reviews Cancer 22, no. 8 (2022): 481–491. 35488036 10.1038/s41568-022-00475-0

[126] M. Schneider , L. Vollmer , A. L. Potthoff , et al., “Meclofenamate Causes Loss of Cellular Tethering and Decoupling of Functional Networks in Glioblastoma,” Neuro‐Oncology 23, no. 11 (2021): 1885–1897. 33864086 10.1093/neuonc/noab092 PMC8563322

[127] P. G. Gritsenko , N. Atlasy , C. E. J. Dieteren , et al., “p120‐Catenin‐Dependent Collective Brain Infiltration by Glioma Cell Networks,” Nature Cell Biology 22, no. 1 (2020): 97–107. 31907411 10.1038/s41556-019-0443-x PMC6952556

[128] F. S. Varn , K. C. Johnson , J. Martinek , et al., “Glioma Progression Is Shaped by Genetic Evolution and Microenvironment Interactions,” Cell 185, no. 12 (2022): 2184–2199.e16. 35649412 10.1016/j.cell.2022.04.038 PMC9189056

[129] T. Yoshida , A. Yamagata , A. Imai , et al., “Canonical Versus Non‐canonical Transsynaptic Signaling of Neuroligin 3 Tunes Development of Sociality in Mice,” Nature Communications 12, no. 1 (2021): 1848. 10.1038/s41467-021-22059-6 PMC7988105 33758193

[130] M. Varghese , N. Keshav , S. Jacot‐Descombes , et al., “Autism Spectrum Disorder: Neuropathology and Animal Models,” Acta Neuropathologica 134, no. 4 (2017): 537–566. 28584888 10.1007/s00401-017-1736-4 PMC5693718

[131] H. Hörnberg , E. Pérez‐Garci , D. Schreiner , et al., “Rescue of Oxytocin Response and Social Behaviour in a Mouse Model of Autism,” Nature 584, no. 7820 (2020): 252–256. 32760004 10.1038/s41586-020-2563-7 PMC7116741

[132] R. Liu , X. P. Qin , Y. Zhuang , et al., “Glioblastoma Recurrence Correlates With NLGN3 Levels,” Cancer Medicine 7, no. 7 (2018): 2848–2859. 29777576 10.1002/cam4.1538 PMC6051187

[133] E. Camporesi , J. Nilsson , A. Vrillon , et al., “Quantification of the Trans‐Synaptic Partners Neurexin‐Neuroligin in CSF of Neurodegenerative Diseases by Parallel Reaction Monitoring Mass Spectrometry,” eBioMedicine 75 (2022): 103793. 34990894 10.1016/j.ebiom.2021.103793 PMC8743209

[134] N.‐N. Dang , X. B. Li , M. Zhang , C. Han , X. Y. Fan , and S. H. Huang , “NLGN3 Upregulates Expression of ADAM10 to Promote the Cleavage of NLGN3 via Activating the LYN Pathway in Human Gliomas,” Frontiers in Cell and Developmental Biology 9 (2021): 662763. 34485271 10.3389/fcell.2021.662763 PMC8415229

[135] P.‐H. Kuhn , A. V. Colombo , B. Schusser , et al., “Systematic Substrate Identification Indicates a Central Role for the Metalloprotease ADAM10 in Axon Targeting and Synapse Function,” eLife 5 (2016): e12748. 26802628 10.7554/eLife.12748 PMC4786429

[136] Y. Wang , Y. Y. Liu , M. B. Chen , et al., “Neuronal‐Driven Glioma Growth Requires Gαi1 and Gαi3,” Theranostics 11, no. 17 (2021): 8535–8549. 34373757 10.7150/thno.61452 PMC8343996

[137] P. Jain , L. F. Surrey , J. Straka , et al., “Novel FGFR2‐INA Fusion Identified in Two Low‐Grade Mixed Neuronal‐Glial Tumors Drives Oncogenesis via MAPK and PI3K/mTOR Pathway Activation,” Acta Neuropathologica 136, no. 1 (2018): 167–169. 29767381 10.1007/s00401-018-1864-5 PMC6015095

[138] S. Luszczak , C. Kumar , V. K. Sathyadevan , et al., “PIM Kinase Inhibition: Co‐Targeted Therapeutic Approaches in Prostate Cancer,” Signal Transduction and Targeted Therapy 5, no. 1 (2020): 7. 32296034 10.1038/s41392-020-0109-y PMC6992635

[139] P. B. Crino , “mTOR: A Pathogenic Signaling Pathway in Developmental Brain Malformations,” Trends in Molecular Medicine 17, no. 12 (2011): 734–742. 21890410 10.1016/j.molmed.2011.07.008

[140] A. A. Ren , D. A. Snellings , Y. S. Su , et al., “PIK3CA and CCM Mutations Fuel Cavernomas Through a Cancer‐Like Mechanism,” Nature 594, no. 7862 (2021): 271–276. 33910229 10.1038/s41586-021-03562-8 PMC8626098

[141] M. Raman , W. Chen , and M. H. Cobb , “Differential Regulation and Properties of MAPKs,” Oncogene 26, no. 22 (2007): 3100–3112. 17496909 10.1038/sj.onc.1210392

[142] X. Deschênes‐Simard , F. Kottakis , S. Meloche , and G. Ferbeyre , “ERKs in Cancer: Friends or Foes?,” Cancer Research 74, no. 2 (2014): 412–419. 24408923 10.1158/0008-5472.CAN-13-2381

[143] H. L. Goel and A. M. Mercurio , “VEGF Targets the Tumour Cell,” Nature Reviews Cancer 13, no. 12 (2013): 871–882. 24263190 10.1038/nrc3627 PMC4011842

[144] E. M. Cherry , D. W. Lee , J. U. Jung , and R. Sitcheran , “Tumor Necrosis Factor‐Like Weak Inducer of Apoptosis (TWEAK) Promotes Glioma Cell Invasion Through Induction of NF‐κB‐Inducing Kinase (NIK) and Noncanonical NF‐κB Signaling,” Molecular Cancer 14, no. 1 (2015): 9. 25622756 10.1186/s12943-014-0273-1 PMC4320546

[145] D. Schmidt , B. Textor , O. T. Pein , et al., “Critical Role for NF‐κB‐Induced JunB in VEGF Regulation and Tumor Angiogenesis,” EMBO Journal 26, no. 3 (2007): 710–719. 17255940 10.1038/sj.emboj.7601539 PMC1794395

[146] E. Varfolomeev , J. W. Blankenship , S. M. Wayson , et al., “IAP Antagonists Induce Autoubiquitination of c‐IAPs, NF‐κB Activation, and TNFα‐Dependent Apoptosis,” Cell 131, no. 4 (2007): 669–681. 18022362 10.1016/j.cell.2007.10.030

[147] J. Liang , Y. Saad , T. Lei , et al., “MCP‐Induced Protein 1 Deubiquitinates TRAF Proteins and Negatively Regulates JNK and NF‐κB Signaling,” Journal of Experimental Medicine 207, no. 13 (2010): 2959–2973. 21115689 10.1084/jem.20092641 PMC3005225

[148] I. E. Wertz , K. M. O'Rourke , H. Zhou , et al., “De‐Ubiquitination and Ubiquitin Ligase Domains of A20 Downregulate NF‐κB Signalling,” Nature 430, no. 7000 (2004): 694–699. 15258597 10.1038/nature02794

[149] P. S. Kumar , A. Shiras , G. das , J. C. Jagtap , V. Prasad , and P. Shastry , “Differential Expression and Role of p21cip/waf1 and p27kip1 in TNF‐α‐Induced Inhibition of Proliferation in Human Glioma Cells,” Molecular Cancer 6, no. 1 (2007): 42. 17565690 10.1186/1476-4598-6-42 PMC1904457

[150] Z. Li , W. Gao , Y. Fei , et al., “NLGN3 Promotes Neuroblastoma Cell Proliferation and Growth Through Activating PI3K/AKT Pathway,” European Journal of Pharmacology 857 (2019): 172423. 31150649 10.1016/j.ejphar.2019.172423

[151] E. J. Yun , D. Kim , S. Kim , J. T. Hsieh , and S. T. Baek , “Targeting Wnt/Beta‐Catenin‐Mediated Upregulation of Oncogenic NLGN3 Suppresses Cancer Stem Cells in Glioblastoma,” Cell Death and Disease 14, no. 7 (2023): 423. 37443071 10.1038/s41419-023-05967-x PMC10344874

[152] J. Li , B. Zhang , Z. Feng , et al., “Stabilization of KPNB1 by Deubiquitinase USP7 Promotes Glioblastoma Progression Through the YBX1‐NLGN3 Axis,” Journal of Experimental and Clinical Cancer Research 43, no. 1 (2024): 28. 38254206 10.1186/s13046-024-02954-8 PMC11040697

[153] H. Nakata and S. Nakamura , “Brain‐Derived Neurotrophic Factor Regulates AMPA Receptor Trafficking to Post‐Synaptic Densities via IP3R and TRPC Calcium Signaling,” FEBS Letters 581, no. 10 (2007): 2047–2054. 17482902 10.1016/j.febslet.2007.04.041

[154] B. W. Luikart , S. Nef , T. Virmani , et al., “TrkB Has a Cell‐Autonomous Role in the Establishment of Hippocampal Schaffer Collateral Synapses,” Journal of Neuroscience 25, no. 15 (2005): 3774–3786. 15829629 10.1523/JNEUROSCI.0041-05.2005 PMC6724922

[155] A. Figurov , L. D. Pozzo‐Miller , P. Olafsson , T. Wang , and B. Lu , “Regulation of Synaptic Responses to High‐Frequency Stimulation and LTP by Neurotrophins in the Hippocampus,” Nature 381, no. 6584 (1996): 706–709. 8649517 10.1038/381706a0

[156] C. A. Chapleau and L. Pozzo‐Miller , “Divergent Roles ofp75NTRand Trk Receptors in BDNF's Effects on Dendritic Spine Density and Morphology,” Neural Plasticity 2012 (2012): 1–9. 10.1155/2012/578057 PMC3323862 22548193

[157] M. Korte , P. Carroll , E. Wolf , G. Brem , H. Thoenen , and T. Bonhoeffer , “Hippocampal Long‐Term Potentiation Is Impaired in Mice Lacking Brain‐Derived Neurotrophic Factor,” Proceedings of the National Academy of Sciences of the United States of America 92, no. 19 (1995): 8856–8860. 7568031 10.1073/pnas.92.19.8856 PMC41066

[158] W. Y. Shen , C. Luo , P. R. Hurtado , et al., “Up‐Regulation of proBDNF/p75(NTR) Signaling in Antibody‐Secreting Cells Drives Systemic Lupus Erythematosus,” Science Advances 8, no. 3 (2022): eabj2797. 35044824 10.1126/sciadv.abj2797 PMC8769540

[159] T. Matsumoto , S. Rauskolb , M. Polack , et al., “Biosynthesis and Processing of Endogenous BDNF: CNS Neurons Store and Secrete BDNF, Not Pro‐BDNF,” Nature Neuroscience 11, no. 2 (2008): 131–133. 18204444 10.1038/nn2038

[160] J. Yang , C. J. Siao , G. Nagappan , et al., “Neuronal Release of proBDNF,” Nature Neuroscience 12, no. 2 (2009): 113–115. 19136973 10.1038/nn.2244 PMC2737352

[161] A. Guemez‐Gamboa , L. Xu , D. Meng , and N. C. Spitzer , “Non‐Cell‐Autonomous Mechanism of Activity‐Dependent Neurotransmitter Switching,” Neuron 82, no. 5 (2014): 1004–1016. 24908484 10.1016/j.neuron.2014.04.029 PMC4072120

[162] H. Yin , Y. Jiang , Y. Zhang , H. Ge , and Z. Yang , “The Inhibition of BDNF/TrkB/PI3K/Akt Signal Mediated by AG1601 Promotes Apoptosis in Malignant Glioma,” Journal of Cellular Biochemistry 120, no. 11 (2019): 18771–18781. 31219215 10.1002/jcb.29190

[163] J. F. Huo and X. B. Chen , “P2X4R Silence Suppresses Glioma Cell Growth Through BDNF/TrkB/ATF4 Signaling Pathway,” Journal of Cellular Biochemistry 120, no. 4 (2019): 6322–6329. 30362154 10.1002/jcb.27919

[164] Y. Tajima , R. P. Molina Jr , L. B. Rorke , et al., “Neurotrophins and Neuronal Versus Glial Differentiation in Medulloblastomas and Other Pediatric Brain Tumors,” Acta Neuropathologica 95, no. 4 (1998): 325–332. 9560008 10.1007/s004010050806

[165] E. Aronica , S. Leenstra , G. H. Jansen , C. W. M. van Veelen , B. Yankaya , and D. Troost , “Expression of Brain‐Derived Neurotrophic Factor and Tyrosine Kinase B Receptor Proteins in Glioneuronal Tumors From Patients With Intractable Epilepsy: Colocalization With N‐Methyl‐D‐Aspartic Acid Receptor,” Acta Neuropathologica 101, no. 4 (2001): 383–392. 11355310 10.1007/s004010000296

[166] V. C. Russo and G. A. Werther , “Des (1‐3) IGF‐I Potently Enhances Differentiated Cell Growth in Olfactory Bulb Organ Culture,” Growth Factors 11, no. 4 (2009): 301–311. 10.3109/08977199409011003 7779409

[167] A. N. Ziegler , S. W. Levison , and T. L. Wood , “Insulin and IGF Receptor Signalling in Neural‐Stem‐Cell Homeostasis,” Nature Reviews Endocrinology 11, no. 3 (2014): 161–170. 10.1038/nrendo.2014.208 PMC5513669 25445849

[168] A. M. Fernandez and I. Torres‐Alemán , “The Many Faces of Insulin‐Like Peptide Signalling in the Brain,” Nature Reviews Neuroscience 13, no. 4 (2012): 225–239. 22430016 10.1038/nrn3209

[169] K. Pandey , B. Bessières , S. L. Sheng , et al., “Neuronal Activity Drives IGF2 Expression From Pericytes to Form Long‐Term Memory,” Neuron 111, no. 23 (2023): 3819–3836.e8. 37788670 10.1016/j.neuron.2023.08.030 PMC10843759

[170] S. Shapira , S. Mathai , R. Zhang , and J. Guan , “Delayed Peripheral Administration of the N‐Terminal Tripeptide of IGF‐1 (GPE) Reduces Brain Damage Following Microsphere Induced Embolic Damage in Young Adult and Aged Rats,” Neuroscience Letters 454, no. 1 (2009): 53–57. 19429053 10.1016/j.neulet.2009.03.003

[171] P. Gupta , D. F. Albeanu , and U. S. Bhalla , “Olfactory Bulb Coding of Odors, Mixtures and Sniffs Is a Linear Sum of Odor Time Profiles,” Nature Neuroscience 18, no. 2 (2015): 272–281. 25581362 10.1038/nn.3913

[172] M. Migliore , F. Cavarretta , A. Marasco , E. Tulumello , M. L. Hines , and G. M. Shepherd , “Synaptic Clusters Function as Odor Operators in the Olfactory Bulb,” Proceedings of the National Academy of Sciences of the United States of America 112, no. 27 (2015): 8499–8504. 26100895 10.1073/pnas.1502513112 PMC4500266

[173] Y. Chen , X. Chen , B. Baserdem , et al., “High‐Throughput Sequencing of Single Neuron Projections Reveals Spatial Organization in the Olfactory Cortex,” Cell 185, no. 22 (2022): 4117–4134.e28. 36306734 10.1016/j.cell.2022.09.038 PMC9681627

[174] A. Alvarez‐Buylla , J. M. Garcia‐Verdugo , and A. D. Tramontin , “A Unified Hypothesis on the Lineage of Neural Stem Cells,” Nature Reviews. Neuroscience 2, no. 4 (2001): 287–293. 11283751 10.1038/35067582

[175] N. S. Roy , S. Wang , L. Jiang , et al., “In Vitro Neurogenesis by Progenitor Cells Isolated From the Adult Human Hippocampus,” Nature Medicine 6, no. 3 (2000): 271–277. 10.1038/73119 10700228

[176] R. Galli , A. Gritti , L. Bonfanti , and A. L. Vescovi , “Neural Stem Cells: An Overview,” Circulation Research 92, no. 6 (2003): 598–608. 12676811 10.1161/01.RES.0000065580.02404.F4

[177] H. Klassen , D. S. Sakaguchi , and M. J. Young , “Stem Cells and Retinal Repair,” Progress in Retinal and Eye Research 23, no. 2 (2004): 149–181. 15094129 10.1016/j.preteyeres.2004.01.002

[178] W. D. Richardson , N. Kessaris , and N. Pringle , “Oligodendrocyte wars,” Nature Reviews. Neuroscience 7, no. 1 (2006): 11–18. 16371946 10.1038/nrn1826 PMC6328010

[179] A. Nishiyama , M. Komitova , R. Suzuki , and X. Zhu , “Polydendrocytes (NG2 Cells): Multifunctional Cells With Lineage Plasticity,” Nature Reviews. Neuroscience 10, no. 1 (2009): 9–22. 19096367 10.1038/nrn2495

[180] J. Buchanan , N. M. da Costa , and L. Cheadle , “Emerging Roles of Oligodendrocyte Precursor Cells in Neural Circuit Development and Remodeling,” Trends in Neurosciences 46, no. 8 (2023): 628–639. 37286422 10.1016/j.tins.2023.05.007 PMC10524797

[181] T. W. Chapman , G. E. Olveda , X. Bame , E. Pereira , and R. A. Hill , “Oligodendrocyte Death Initiates Synchronous Remyelination to Restore Cortical Myelin Patterns in Mice,” Nature Neuroscience 26, no. 4 (2023): 555–569. 36928635 10.1038/s41593-023-01271-1 PMC10208560

[182] M. Monje , S. S. Mitra , M. E. Freret , et al., “Hedgehog‐Responsive Candidate Cell of Origin for Diffuse Intrinsic Pontine Glioma,” Proceedings of the National Academy of Sciences of the United States of America 108, no. 11 (2011): 4453–4458. 21368213 10.1073/pnas.1101657108 PMC3060250

[183] C. Neftel , J. Laffy , M. G. Filbin , et al., “An Integrative Model of Cellular States, Plasticity, and Genetics for Glioblastoma,” Cell 178, no. 4 (2019): 835–849.e21. 31327527 10.1016/j.cell.2019.06.024 PMC6703186

[184] I. Liu , L. Jiang , E. R. Samuelsson , et al., “The Landscape of Tumor Cell States and Spatial Organization in H3‐K27M Mutant Diffuse Midline Glioma Across Age and Location,” Nature Genetics 54, no. 12 (2022): 1881–1894. 36471067 10.1038/s41588-022-01236-3 PMC9729116

[185] C. Liu , J. C. Sage , M. R. Miller , et al., “Mosaic Analysis With Double Markers Reveals Tumor Cell of Origin in Glioma,” Cell 146, no. 2 (2011): 209–221. 21737130 10.1016/j.cell.2011.06.014 PMC3143261

[186] M. W. Vondran , P. Clinton‐Luke , J. Z. Honeywell , and C. F. Dreyfus , “BDNF+/− Mice Exhibit Deficits in Oligodendrocyte Lineage Cells of the Basal Forebrain,” Glia 58, no. 7 (2010): 848–856. 20091777 10.1002/glia.20969 PMC2851835

[187] A. W. Wong , J. Xiao , D. Kemper , T. J. Kilpatrick , and S. S. Murray , “Oligodendroglial Expression of TrkB Independently Regulates Myelination and Progenitor Cell Proliferation,” Journal of Neuroscience 33, no. 11 (2013): 4947–4957. 23486965 10.1523/JNEUROSCI.3990-12.2013 PMC6619007

[188] S. Jessa , A. Mohammadnia , A. S. Harutyunyan , et al., “K27M in Canonical and Noncanonical H3 Variants Occurs in Distinct Oligodendroglial Cell Lineages in Brain Midline Gliomas,” Nature Genetics 54, no. 12 (2022): 1865–1880. 36471070 10.1038/s41588-022-01205-w PMC9742294

[189] D. E. Bergles , J. D. B. Roberts , P. Somogyi , and C. E. Jahr , “Glutamatergic Synapses on Oligodendrocyte Precursor Cells in the Hippocampus,” Nature 405, no. 6783 (2000): 187–191. 10821275 10.1038/35012083

[190] C. W. Mount , B. Yalçın , K. Cunliffe‐Koehler , S. Sundaresh , and M. Monje , “Monosynaptic Tracing Maps Brain‐Wide Afferent Oligodendrocyte Precursor Cell Connectivity,” eLife 8 (2019): e49291. 31625910 10.7554/eLife.49291 PMC6800000

[191] D. Schiff , H. Messersmith , P. K. Brastianos , et al., “Radiation Therapy for Brain Metastases: ASCO Guideline Endorsement of ASTRO Guideline,” Journal of Clinical Oncology 40, no. 20 (2022): 2271–2276. 35561283 10.1200/JCO.22.00333

[192] P. Fatemi , M. Zhang , K. J. Miller , P. Robe , and G. Li , “How Intraoperative Tools and Techniques Have Changed the Approach to Brain Tumor Surgery,” Current Oncology Reports 20, no. 11 (2018): 89. 30259202 10.1007/s11912-018-0723-9

[193] J. H. Sampson , M. D. Gunn , P. E. Fecci , and D. M. Ashley , “Brain Immunology and Immunotherapy in Brain Tumours,” Nature Reviews Cancer 20, no. 1 (2019): 12–25. 31806885 10.1038/s41568-019-0224-7 PMC7327710

[194] J. H. Suh , R. Kotecha , S. T. Chao , M. S. Ahluwalia , A. Sahgal , and E. L. Chang , “Current Approaches to the Management of Brain Metastases,” Nature Reviews Clinical Oncology 17, no. 5 (2020): 279–299. 10.1038/s41571-019-0320-3 32080373

[195] Y. Odia , A. N. Gutierrez , and R. Kotecha , “Surgically Targeted Radiation Therapy (STaRT) Trials for Brain Neoplasms: A Comprehensive Review,” Neuro‐Oncology 24, no. Supplement_6 (2022): S16–S24. 36322100 10.1093/neuonc/noac130 PMC9629486

[196] L. Pang , F. Khan , M. Dunterman , and P. Chen , “Pharmacological Targeting of the Tumor–Immune Symbiosis in Glioblastoma,” Trends in Pharmacological Sciences 43, no. 8 (2022): 686–700. 35534356 10.1016/j.tips.2022.04.002 PMC9288491

[197] L. Rong , N. Li , and Z. Zhang , “Emerging Therapies for Glioblastoma: Current State and Future Directions,” Journal of Experimental and Clinical Cancer Research 41, no. 1 (2022): 142. 35428347 10.1186/s13046-022-02349-7 PMC9013078

[198] S. Li , C. Wang , J. Chen , et al., “Signaling Pathways in Brain Tumors and Therapeutic Interventions,” Signal Transduction and Targeted Therapy 8, no. 1 (2023): 8. 36596785 10.1038/s41392-022-01260-z PMC9810702

[199] K. R. Noll , H. S. Chen , J. S. Wefel , et al., “Alterations in Functional Connectomics Associated With Neurocognitive Changes Following Glioma Resection,” Neurosurgery 88, no. 3 (2021): 544–551. 33080024 10.1093/neuros/nyaa453 PMC7884148

[200] D. Gramatzki , J. Felsberg , P. Roth , et al., “The Molecular Evolution of Glioblastoma Treated by Gross Total Resection Alone,” Neuro‐Oncology 23, no. 2 (2021): 334–336. 33173940 10.1093/neuonc/noaa261 PMC7906049

[201] S. Hu , H. Kang , Y. Baek , G. el Fakhri , A. Kuang , and H. S. Choi , “Real‐Time Imaging of Brain Tumor for Image‐Guided Surgery,” Advanced Healthcare Materials 7, no. 16 (2018): e1800066. 29719137 10.1002/adhm.201800066 PMC6105507

[202] A. H. D. Silva and K. Aquilina , “Surgical Approaches in Pediatric Neuro‐Oncology,” Cancer and Metastasis Reviews 38, no. 4 (2019): 723–747. 31863234 10.1007/s10555-019-09832-2

[203] A. Wu , J. Y. Wu , and M. Lim , “Updates in Intraoperative Strategies for Enhancing Intra‐Axial Brain Tumor Control,” Neuro‐Oncology 24, no. Supplement_6 (2022): S33–S41. 36322098 10.1093/neuonc/noac170 PMC9629479

[204] N. J. Laperriere and M. Bernstein , “Radiotherapy for Brain Tumors,” CA: A Cancer Journal for Clinicians 44, no. 2 (1994): 96–108. 8124609 10.3322/canjclin.44.2.96

[205] C. Chargari , F. Campana , J. Y. Pierga , et al., “Whole‐Brain Radiation Therapy in Breast Cancer Patients With Brain Metastases,” Nature Reviews Clinical Oncology 7, no. 11 (2010): 632–640. 10.1038/nrclinonc.2010.119 20625374

[206] S. Perkins and S. Acharya , “Radiation Therapy to the Developing Brain: Advanced Technology Is Ready for Robust Optimization Parameters,” Neuro‐Oncology 23, no. 3 (2021): 350–351. 33560406 10.1093/neuonc/noab007 PMC7992876

[207] A. Shergalis , A. Bankhead, III , U. Luesakul , N. Muangsin , and N. Neamati , “Current Challenges and Opportunities in Treating Glioblastoma,” Pharmacological Reviews 70, no. 3 (2018): 412–445. 29669750 10.1124/pr.117.014944 PMC5907910

[208] S. Crunkhorn , “Targeting drug‐resistant glioblastoma,” Nature Reviews Drug Discovery 21, no. 10 (2022): 711. 10.1038/d41573-022-00146-7 36045286

[209] V. Venkataramani , D. I. Tanev , T. Kuner , W. Wick , and F. Winkler , “Synaptic Input to Brain Tumors: Clinical Implications,” Neuro‐Oncology 23, no. 1 (2021): 23–33. 32623467 10.1093/neuonc/noaa158 PMC7850064

[210] A. O. Rossetti , S. Jeckelmann , J. Novy , P. Roth , M. Weller , and R. Stupp , “Levetiracetam and Pregabalin for Antiepileptic Monotherapy in Patients With Primary Brain Tumors. A Phase II Randomized Study,” Neuro‐Oncology 16, no. 4 (2013): 584–588. 24311644 10.1093/neuonc/not170 PMC3956345

[211] J. M. Margolis , B. C. Chu , Z. J. Wang , R. Copher , and J. E. Cavazos , “Effectiveness of Antiepileptic Drug Combination Therapy for Partial‐Onset Seizures Based on Mechanisms of Action,” JAMA Neurology 71, no. 8 (2014): 985–993. 24911669 10.1001/jamaneurol.2014.808

[212] G. Huberfeld and C. J. Vecht , “Seizures and Gliomas — Towards a Single Therapeutic Approach,” Nature Reviews Neurology 12, no. 4 (2016): 204–216. 26965673 10.1038/nrneurol.2016.26

[213] M. A. Francisco , S. Wanggou , J. J. Fan , et al., “Chloride Intracellular Channel 1 Cooperates With Potassium Channel EAG2 to Promote Medulloblastoma Growth,” Journal of Experimental Medicine 217, no. 5 (2020): e20190971. 32097463 10.1084/jem.20190971 PMC7201926

[214] E. Sokolov , J. Dietrich , and A. J. Cole , “The Complexities Underlying Epilepsy in People With Glioblastoma,” Lancet Neurology 22, no. 6 (2023): 505–516. 37121239 10.1016/S1474-4422(23)00031-5

[215] C. A. Sanchez Trivino , R. Spelat , F. Spada , et al., “Exosomal TNF‐α Mediates Voltage‐Gated Na+ Channels 1.6 Overexpression and Contributes to Brain‐Tumor Induced Neuronal Hyperexcitability,” Journal of Clinical Investigation 134 (2024): e166271. 39088270 10.1172/JCI166271 PMC11405049

[216] M. T. Kronschläger , R. Drdla‐Schutting , M. Gassner , S. D. Honsek , H. L. Teuchmann , and J. Sandkühler , “Gliogenic LTP Spreads Widely in Nociceptive Pathways,” Science 354, no. 6316 (2016): 1144–1148. 27934764 10.1126/science.aah5715 PMC6145441

[217] F. Gambino , S. Pagès , V. Kehayas , et al., “Sensory‐Evoked LTP Driven by Dendritic Plateau Potentials In Vivo,” Nature 515, no. 7525 (2014): 116–119. 25174710 10.1038/nature13664

[218] B. Li , B. S. Suutari , S.(.) D. Sun , et al., “Neuronal Inactivity co‐Opts LTP Machinery to Drive Potassium Channel Splicing and Homeostatic Spike Widening,” Cell 181, no. 7 (2020): 1547–1565.e15. 32492405 10.1016/j.cell.2020.05.013 PMC9310388

[219] B. Yang , J. Sanches‐Padilla , J. Kondapalli , et al., “Locus Coeruleus Anchors a Trisynaptic Circuit Controlling Fear‐Induced Suppression of Feeding,” Neuron 109, no. 5 (2021): 823–838.e6. 33476548 10.1016/j.neuron.2020.12.023 PMC9272546

[220] S. Marwaha , E. Palmer , T. Suppes , E. Cons , A. H. Young , and R. Upthegrove , “Novel and Emerging Treatments for Major Depression,” Lancet 401, no. 10371 (2023): 141–153. 36535295 10.1016/S0140-6736(22)02080-3

[221] I. K. Mellinghoff , M. Lu , P. Y. Wen , et al., “Vorasidenib and Ivosidenib in IDH1‐Mutant Low‐Grade Glioma: A Randomized, Perioperative Phase 1 Trial,” Nature Medicine 29, no. 3 (2023): 615–622. 10.1038/s41591-022-02141-2 PMC10313524 36823302

[222] L. Qin , Z. Liu , S. Guo , et al., “Astrocytic Neuroligin‐3 Influences Gene Expression and Social Behavior, But Is Dispensable for Synapse Number,” Molecular Psychiatry (2024): 1–13. 39003414 10.1038/s41380-024-02659-6 PMC11649564

[223] L. R. Gauthier , B. C. Charrin , M. Borrell‐Pagès , et al., “Huntingtin Controls Neurotrophic Support and Survival of Neurons by Enhancing BDNF Vesicular Transport Along Microtubules,” Cell 118, no. 1 (2004): 127–138. 15242649 10.1016/j.cell.2004.06.018

[224] L. Minichiello , “TrkB Signalling Pathways in LTP and Learning,” Nature Reviews. Neuroscience 10, no. 12 (2009): 850–860. 19927149 10.1038/nrn2738

[225] K. Zhang , F. Wang , M. Zhai , et al., “Hyperactive Neuronal Autophagy Depletes BDNF and Impairs Adult Hippocampal Neurogenesis in a Corticosterone‐Induced Mouse Model of Depression,” Theranostics 13, no. 3 (2023): 1059–1075. 36793868 10.7150/thno.81067 PMC9925310

[226] S. F. Murphy , R. T. Varghese , S. Lamouille , et al., “Connexin 43 Inhibition Sensitizes Chemoresistant Glioblastoma Cells to Temozolomide,” Cancer Research 76, no. 1 (2016): 139–149. 26542214 10.1158/0008-5472.CAN-15-1286 PMC5113032

[227] A.‐L. Potthoff , D. H. Heiland , B. O. Evert , et al., “Inhibition of Gap Junctions Sensitizes Primary Glioblastoma Cells for Temozolomide,” Cancers 11, no. 6 (2019): 858. 31226836 10.3390/cancers11060858 PMC6628126

[228] J. L. Munoz , V. Rodriguez‐Cruz , S. J. Greco , S. H. Ramkissoon , K. L. Ligon , and P. Rameshwar , “Temozolomide Resistance in Glioblastoma Cells Occurs Partly Through Epidermal Growth Factor Receptor‐Mediated Induction of Connexin 43,” Cell Death and Disease 5, no. 3 (2014): e1145. 24675463 10.1038/cddis.2014.111 PMC3973225

[229] M. Schneider , A. L. Potthoff , B. O. Evert , et al., “Inhibition of Intercellular Cytosolic Traffic Via Gap Junctions Reinforces Lomustine‐Induced Toxicity in Glioblastoma Independent of MGMT Promoter Methylation Status,” Pharmaceuticals 14, no. 3 (2021): 195. 33673490 10.3390/ph14030195 PMC7997332

[230] Y. Zhang , Y. Wen , J. Nie , et al., “MYEF2: An Immune Infiltration‐Related Prognostic Factor in IDH‐Wild‐Type Glioblastoma,” Aging 15, no. 15 (2023): 7760–7780. 37556355 10.18632/aging.204939 PMC10457068

[231] P. Matarrese , M. Signore , B. Ascione , G. Fanelli , M. G. Paggi , and C. Abbruzzese , “Chlorpromazine Overcomes Temozolomide Resistance in Glioblastoma by Inhibiting Cx43 and Essential DNA Repair Pathways,” Journal of Translational Medicine 22, no. 1 (2024): 667. 39026284 10.1186/s12967-024-05501-3 PMC11256652

[232] C.‐H. Lee , Y.‐W. Cheng , and G. S. Huang , “Topographical Control of Cell‐Cell Interaction in C6 Glioma by Nanodot Arrays,” Nanoscale Research Letters 9, no. 1 (2014): 250. 24917700 10.1186/1556-276X-9-250 PMC4032869

[233] D. C. Watson , D. Bayik , S. Storevik , et al., “GAP43‐Dependent Mitochondria Transfer From Astrocytes Enhances Glioblastoma Tumorigenicity,” Nature Cancer 4, no. 5 (2023): 648–664. 37169842 10.1038/s43018-023-00556-5 PMC10212766

[234] P. Müller , D. Dietrich , S. Schoch , J. Pitsch , A. J. Becker , and S. Cases‐Cunillera , “Ganglioglioma Cells Potentiate Neuronal Network Synchronicity and Elicit Burst Discharges via Released Factors,” Neurobiology of Disease 190 (2024): 106364. 38008342 10.1016/j.nbd.2023.106364

[235] A. Krigers , M. Demetz , P. Moser , et al., “Impact of GAP‐43, Cx43 and Actin Expression on the Outcome and Overall Survival in Diffuse and Anaplastic Gliomas,” Scientific Reports 13, no. 1 (2023): 2024. 36739296 10.1038/s41598-023-29298-1 PMC9899260

[236] Z. He , J. Dai , J. D. L. Ho , et al., “Interactive Multi‐Stage Robotic Positioner for Intra‐Operative MRI‐Guided Stereotactic Neurosurgery,” Advanced Science 11, no. 7 (2023): 2305495. 38072667 10.1002/advs.202305495 PMC10870025

[237] X.‐Q. Lu , A. Mahadevan , G. Mathiowitz , et al., “Frameless Angiogram‐Based Stereotactic Radiosurgery for Treatment of Arteriovenous Malformations,” International Journal of Radiation Oncology, Biology, Physics 84, no. 1 (2012): 274–282. 22284685 10.1016/j.ijrobp.2011.10.044

[238] M. Wang , Y. Zhang , W. Shi , R. Zhu , H. Li , and R. Zhao , “Frameless Robot‐Assisted Stereotactic Biopsy: An Effective and Minimally Invasive Technique for Pediatric Diffuse Intrinsic Pontine Gliomas,” Journal of Neuro‐Oncology 160, no. 1 (2022): 107–114. 35997920 10.1007/s11060-022-04122-4

[239] S. Koizumi , Y. Shiraishi , I. Makita , M. Kadowaki , T. Sameshima , and K. Kurozumi , “A Novel Technique for Fence‐Post Tube Placement in Glioma Using the Robot‐Guided Frameless Neuronavigation Technique Under Exoscope Surgery: Patient Series,” Journal of Neurosurgery: Case Lessons 2, no. 24 (2021): CASE21466. 35855488 10.3171/CASE21466 PMC9281438

[240] Z. He , Y. N. Zhu , Y. Chen , et al., “A Deep Unrolled Neural Network for Real‐Time MRI‐Guided Brain Intervention,” Nature Communications 14, no. 1 (2023): 8257. 10.1038/s41467-023-43966-w PMC10716161 38086851

[241] H. Chen , J. Marino , A. B. Stemer , I. P. Singh , and M. T. Froehler , “Emerging Subspecialties in Neurology: Interventional Neurology,” Neurology 101, no. 19 (2023): e1939–e1942. 37652702 10.1212/WNL.0000000000207821 PMC10663010

[242] Y. Fan , S. Liu , E. Gao , et al., “The LMIT: Light‐Mediated Minimally‐Invasive Theranostics in Oncology,” Theranostics 14, no. 1 (2024): 341–362. 38164160 10.7150/thno.87783 PMC10750201

[243] W. G. Meissner , P. Remy , C. Giordana , et al., “Trial of Lixisenatide in Early Parkinson's Disease,” New England Journal of Medicine 390, no. 13 (2024): 1176–1185. 38598572 10.1056/NEJMoa2312323

[244] B. Balogh , M. Ivánczi , B. Nizami , T. Beke‐Somfai , and I. M. Mándity , “ConjuPepDB: A Database of Peptide–Drug Conjugates,” Nucleic Acids Research 49, no. D1 (2021): D1102–D1112. 33125057 10.1093/nar/gkaa950 PMC7778964

[245] Z. Wang , Y. Zhao , Y. Hou , et al., “A Thrombin‐Activated Peptide‐Templated Nanozyme for Remedying Ischemic Stroke Via Thrombolytic and Neuroprotective Actions,” Advanced Materials 36, no. 10 (2023): e2210144. 36730098 10.1002/adma.202210144

[246] S. Jin , L. Zhang , and L. Wang , “Kaempferol, A Potential Neuroprotective Agent in Neurodegenerative Diseases: From Chemistry to Medicine,” Biomedicine and Pharmacotherapy 165 (2023): 115215. 37494786 10.1016/j.biopha.2023.115215

[247] Q. Sun , Y. Wang , L. Hou , et al., “Clozapine‐N‐Oxide Protects Dopaminergic Neurons Against Rotenone‐Induced Neurotoxicity by Preventing Ferritinophagy‐Mediated Ferroptosis,” Free Radical Biology and Medicine 212 (2024): 384–402. 38182072 10.1016/j.freeradbiomed.2023.12.045 PMC10842931

[248] Y. Wei , M. Lu , M. Mei , et al., “Pyridoxine Induces Glutathione Synthesis Via PKM2‐Mediated Nrf2 Transactivation and Confers Neuroprotection,” Nature Communications 11, no. 1 (2020): 941. 10.1038/s41467-020-14788-x PMC7029000 32071304

[249] S. F. Nabavi , H. Khan , G. D'onofrio , et al., “Apigenin as Neuroprotective Agent: Of Mice and Men,” Pharmacological Research 128 (2018): 359–365. 29055745 10.1016/j.phrs.2017.10.008

[250] A. Wellenberg , V. Brinkmann , J. Bornhorst , N. Ventura , S. Honnen , and G. Fritz , “Cisplatin‐Induced Neurotoxicity Involves the Disruption of Serotonergic Neurotransmission,” Pharmacological Research 174 (2021): 105921. 34601079 10.1016/j.phrs.2021.105921

[251] D. Briukhovetska , J. Dörr , S. Endres , P. Libby , C. A. Dinarello , and S. Kobold , “Interleukins in Cancer: From Biology to Therapy,” Nature Reviews Cancer 21, no. 8 (2021): 481–499. 34083781 10.1038/s41568-021-00363-z PMC8173513

[252] S. Li , B. Mirlekar , B. M. Johnson , et al., “STING‐Induced Regulatory B Cells Compromise NK Function in Cancer Immunity,” Nature 610, no. 7931 (2022): 373–380. 36198789 10.1038/s41586-022-05254-3 PMC9875944

[253] D. J. Propper and F. R. Balkwill , “Harnessing Cytokines and Chemokines for Cancer Therapy,” Nature Reviews Clinical Oncology 19, no. 4 (2022): 237–253. 10.1038/s41571-021-00588-9 34997230

[254] T. R. Mempel , J. K. Lill , and L. M. Altenburger , “How Chemokines Organize the Tumour Microenvironment,” Nature Reviews Cancer 24, no. 1 (2023): 28–50. 38066335 10.1038/s41568-023-00635-w PMC11480775

[255] S. Muhammad , T. Fan , Y. Hai , Y. Gao , and J. He , “Reigniting Hope in Cancer Treatment: The Promise and Pitfalls of IL‐2 and IL‐2R Targeting Strategies,” Molecular Cancer 22, no. 1 (2023): 121. 37516849 10.1186/s12943-023-01826-7 PMC10385932

[256] G. W. Davis , B. Eaton , and S. Paradis , “Synapse Formation Revisited,” Nature Neuroscience 4, no. 6 (2001): 558–560. 11369932 10.1038/88370

[257] N. Toni , E. M. Teng , E. A. Bushong , et al., “Synapse Formation on Neurons Born in the Adult Hippocampus,” Nature Neuroscience 10, no. 6 (2007): 727–734. 17486101 10.1038/nn1908

[258] B. Katz and R. Miledi , “Input‐Output Relation of a Single Synapse,” Nature 212, no. 5067 (1966): 1242–1245. 21090453 10.1038/2121242a0

[259] S. Cohen‐Cory , “The Developing Synapse: Construction and Modulation of Synaptic Structures and Circuits,” Science 298, no. 5594 (2002): 770–776. 12399577 10.1126/science.1075510

[260] M. Zeng , X. Chen , D. Guan , et al., “Reconstituted Postsynaptic Density as a Molecular Platform for Understanding Synapse Formation and Plasticity,” Cell 174, no. 5 (2018): 1172–1187.e16. 30078712 10.1016/j.cell.2018.06.047

[261] X. Chen , B. Jia , Y. Araki , et al., “Arc Weakens Synapses by Dispersing AMPA Receptors From Postsynaptic Density via Modulating PSD Phase Separation,” Cell Research 32, no. 10 (2022): 914–930. 35856091 10.1038/s41422-022-00697-9 PMC9525282

[262] L. Wang , K. Pang , L. Zhou , et al., “A Cross‐Species Proteomic Map Reveals Neoteny of Human Synapse Development,” Nature 622, no. 7981 (2023): 112–119. 37704727 10.1038/s41586-023-06542-2 PMC10576238

[263] O. Awolaran , S. A. Brooks , and V. Lavender , “Breast Cancer Osteomimicry and Its Role in Bone Specific Metastasis; an Integrative, Systematic Review of Preclinical Evidence,” Breast 30 (2016): 156–171. 27750106 10.1016/j.breast.2016.09.017

[264] A. G. Ording , U. Heide‐Jørgensen , C. F. Christiansen , M. Nørgaard , J. Acquavella , and H. T. Sørensen , “Site of Metastasis and Breast Cancer Mortality: A Danish Nationwide Registry‐Based Cohort Study,” Clinical and Experimental Metastasis 34, no. 1 (2016): 93–101. 27718076 10.1007/s10585-016-9824-8

[265] X. Jin , Z. Demere , K. Nair , et al., “A Metastasis Map of Human Cancer Cell Lines,” Nature 588, no. 7837 (2020): 331–336. 33299191 10.1038/s41586-020-2969-2 PMC8439149

[266] F. Andre , T. Filleron , M. Kamal , et al., “Genomics to Select Treatment for Patients With Metastatic Breast Cancer,” Nature 610, no. 7931 (2022): 343–348. 36071165 10.1038/s41586-022-05068-3

[267] Z. Diamantopoulou , F. Castro‐Giner , F. D. Schwab , et al., “The Metastatic Spread of Breast Cancer Accelerates During Sleep,” Nature 607, no. 7917 (2022): 156–162. 35732738 10.1038/s41586-022-04875-y

[268] D. C. Rigiracciolo , M. F. Santolla , R. Lappano , et al., “Focal Adhesion Kinase (FAK) Activation by Estrogens Involves GPER in Triple‐Negative Breast Cancer Cells,” Journal of Experimental and Clinical Cancer Research 38, no. 1 (2019): 58. 30728047 10.1186/s13046-019-1056-8 PMC6364402

[269] R. Deng , H. L. Zhang , J. H. Huang , et al., “MAPK1/3 Kinase‐Dependent ULK1 Degradation Attenuates Mitophagy and Promotes Breast Cancer Bone Metastasis,” Autophagy 17, no. 10 (2020): 3011–3029. 33213267 10.1080/15548627.2020.1850609 PMC8526010

[270] G. Liang , Y. Ling , M. Mehrpour , et al., “Autophagy‐Associated circRNA circCDYL Augments Autophagy and Promotes Breast Cancer Progression,” Molecular Cancer 19, no. 1 (2020): 65. 32213200 10.1186/s12943-020-01152-2 PMC7093993

[271] L. Mao , Y. Y. Zhan , B. Wu , et al., “ULK1 Phosphorylates Exo70 to Suppress Breast Cancer Metastasis,” Nature Communications 11, no. 1 (2020): 117. 10.1038/s41467-019-13923-7 PMC6949295 31913283

[272] R. Xu , H. Tan , J. Zhang , Z. Yuan , Q. Xie , and L. Zhang , “Fam20C in Human Diseases: Emerging Biological Functions and Therapeutic Implications,” Frontiers in Molecular Biosciences 8 (2021): 790172. 34988120 10.3389/fmolb.2021.790172 PMC8721277

[273] R. Zhao , L. Fu , Z. Yuan , et al., “Discovery of a Novel Small‐Molecule Inhibitor of Fam20C That Induces Apoptosis and Inhibits Migration in Triple Negative Breast Cancer,” European Journal of Medicinal Chemistry 210 (2021): 113088. 33316691 10.1016/j.ejmech.2020.113088

[274] H. Zuo , D. Yang , and Y. Wan , “Fam20C Regulates Bone Resorption and Breast Cancer Bone Metastasis Through Osteopontin and BMP4,” Cancer Research 81, no. 20 (2021): 5242–5254. 34433585 10.1158/0008-5472.CAN-20-3328

[275] Y.‐Y. Liu , H. Y. Liu , T. J. Yu , et al., “O‐GlcNAcylation of MORC2 at Threonine 556 by OGT Couples TGF‐β Signaling to Breast Cancer Progression,” Cell Death and Differentiation 29, no. 4 (2022): 861–873. 34974534 10.1038/s41418-021-00901-0 PMC8991186

[276] D. K. Yadav , A. Sharma , P. Dube , S. Shaikh , H. Vaghasia , and R. M. Rawal , “Identification of Crucial Hub Genes and Potential Molecular Mechanisms in Breast Cancer by Integrated Bioinformatics Analysis and Experimental Validation,” Computers in Biology and Medicine 149 (2022): 106036. 36096037 10.1016/j.compbiomed.2022.106036

[277] S. Gawrzak , L. Rinaldi , S. Gregorio , et al., “MSK1 Regulates Luminal Cell Differentiation and Metastatic Dormancy in ER(+) Breast Cancer,” Nature Cell Biology 20, no. 2 (2018): 211–221. 29358704 10.1038/s41556-017-0021-z

[278] Q. Wu , P. Tian , D. He , et al., “SCUBE2 Mediates Bone Metastasis of Luminal Breast Cancer by Modulating Immune‐Suppressive Osteoblastic Niches,” Cell Research 33, no. 6 (2023): 464–478. 37142671 10.1038/s41422-023-00810-6 PMC10235122

[279] V. S. Tagliabracci , J. L. Engel , J. Wen , et al., “Secreted Kinase Phosphorylates Extracellular Proteins That Regulate Biomineralization,” Science 336, no. 6085 (2012): 1150–1153. 22582013 10.1126/science.1217817 PMC3754843

[280] T. Koike , T. Mikami , J. I. Tamura , and H. Kitagawa , “Altered Sulfation Status of FAM20C‐Dependent Chondroitin Sulfate Is Associated With Osteosclerotic Bone Dysplasia,” Nature Communications 13, no. 1 (2022): 7952. 10.1038/s41467-022-35687-3 PMC9792594 36572689

[281] M. Dzamukova , T. M. Brunner , J. Miotla‐Zarebska , et al., “Mechanical Forces Couple Bone Matrix Mineralization With Inhibition of Angiogenesis to Limit Adolescent Bone Growth,” Nature Communications 13, no. 1 (2022): 3059. 10.1038/s41467-022-30618-8 PMC9160028 35650194

[282] R. Wang , K. Chadalavada , J. Wilshire , et al., “Glioblastoma Stem‐Like Cells Give Rise to Tumour Endothelium,” Nature 468, no. 7325 (2010): 829–833. 21102433 10.1038/nature09624

[283] G. M. Marshall , D. R. Carter , B. B. Cheung , et al., “The Prenatal Origins of Cancer,” Nature Reviews. Cancer 14, no. 4 (2014): 277–289. 24599217 10.1038/nrc3679 PMC4041218

[284] M. Yao , S. Li , X. Wu , et al., “Cellular Origin of Glioblastoma and Its Implication in Precision Therapy,” Cellular and Molecular Immunology 15, no. 8 (2018): 737–739. 29553137 10.1038/cmi.2017.159 PMC6141605

[285] Y. Ren , Z. Huang , L. Zhou , et al., “Spatial Transcriptomics Reveals Niche‐Specific Enrichment and Vulnerabilities of Radial Glial Stem‐Like Cells in Malignant Gliomas,” Nature Communications 14, no. 1 (2023): 1028. 10.1038/s41467-023-36707-6 PMC9950149 36823172

[286] A. Xiao , H. Wu , P. P. Pandolfi , D. N. Louis , and T. van Dyke , “Astrocyte Inactivation of the pRb Pathway Predisposes Mice to Malignant Astrocytoma Development That Is Accelerated by PTEN Mutation,” Cancer Cell 1, no. 2 (2002): 157–168. 12086874 10.1016/s1535-6108(02)00029-6

[287] J. H. Chen , P. Zhang , W. D. Chen , et al., “ATM‐Mediated PTEN Phosphorylation Promotes PTEN Nuclear Translocation and Autophagy in Response to DNA‐Damaging Agents in Cancer Cells,” Autophagy 11, no. 2 (2015): 239–252. 25701194 10.1080/15548627.2015.1009767 PMC4502816

[288] K. Funato , T. Major , P. W. Lewis , C. D. Allis , and V. Tabar , “Use of Human Embryonic Stem Cells to Model Pediatric Gliomas With H3.3K27M Histone Mutation,” Science 346, no. 6216 (2014): 1529–1533. 25525250 10.1126/science.1253799 PMC4995593

[289] L. Li , A. Dutra , E. Pak , et al., “EGFRvIII Expression and PTEN Loss Synergistically Induce Chromosomal Instability and Glial Tumors,” Neuro‐Oncology 11, no. 1 (2009): 9–21. 18812521 10.1215/15228517-2008-081 PMC2718963

[290] M. C. Havrda , B. R. Paolella , C. Ran , et al., “Id2 Mediates Oligodendrocyte Precursor Cell Maturation Arrest and Is Tumorigenic in a PDGF‐Rich Microenvironment,” Cancer Research 74, no. 6 (2014): 1822–1832. 24425046 10.1158/0008-5472.CAN-13-1839 PMC5061294

[291] Y. Sun , W. Zhang , D. Chen , et al., “A Glioma Classification Scheme Based on Coexpression Modules of EGFR and PDGFRA,” Proceedings of the National Academy of Sciences of the United States of America 111, no. 9 (2014): 3538–3543. 24550449 10.1073/pnas.1313814111 PMC3948229

[292] P. P. Gonzalez , J. Kim , R. P. Galvao , N. Cruickshanks , R. Abounader , and H. Zong , “p53 and NF 1 Loss Plays Distinct but Complementary Roles in Glioma Initiation and Progression,” Glia 66, no. 5 (2018): 999–1015. 29392777 10.1002/glia.23297 PMC7808243

[293] E. S. Jecrois , W. Zheng , M. Bornhorst , et al., “Treatment During a Developmental Window Prevents NF1‐Associated Optic Pathway Gliomas by Targeting Erk‐Dependent Migrating Glial Progenitors,” Developmental Cell 56, no. 20 (2021): 2871–2885.e6. 34428430 10.1016/j.devcel.2021.08.004

[294] R. Verma , X. Chen , D. Xin , et al., “Olig1/2‐Expressing Intermediate Lineage Progenitors Are Predisposed to PTEN/p53‐Loss‐Induced Gliomagenesis and Harbor Specific Therapeutic Vulnerabilities,” Cancer Research 83, no. 6 (2023): 890–905. 36634201 10.1158/0008-5472.CAN-22-1577

[295] A. Wu , M. N. Pangalos , S. Efthimiopoulos , J. Shioi , and N. K. Robakis , “Appican Expression Induces Morphological Changes in C6 Glioma Cells and Promotes Adhesion of Neural Cells to the Extracellular Matrix,” Journal of Neuroscience 17, no. 13 (1997): 4987–4993. 9185536 10.1523/JNEUROSCI.17-13-04987.1997 PMC6573314

[296] H. S. Venkatesh , “The Neural Regulation of Cancer,” Science 366, no. 6468 (2019): 965. 10.1126/science.aaz7776 31753994

[297] P. Dirks , “Bmi1 and Cell of Origin Determinants of Brain Tumor Phenotype,” Cancer Cell 12, no. 4 (2007): 295–297. 17936553 10.1016/j.ccr.2007.10.003

[298] N. A. Charles , E. C. Holland , R. Gilbertson , R. Glass , and H. Kettenmann , “The Brain Tumor Microenvironment,” Glia 59, no. 8 (2011): 1169–1180. 21446047 10.1002/glia.21136

[299] S. Soleman , M. A. Filippov , A. Dityatev , and J. W. Fawcett , “Targeting the Neural Extracellular Matrix in Neurological Disorders,” Neuroscience 253 (2013): 194–213. 24012743 10.1016/j.neuroscience.2013.08.050

[300] F. J. Swartling , S. Bolin , J. J. Phillips , and A. I. Persson , “Signals That Regulate the Oncogenic Fate of Neural Stem Cells and Progenitors,” Experimental Neurology 260 (2014): 56–68. 23376224 10.1016/j.expneurol.2013.01.027 PMC3758390

[301] M. Tang , Q. Xie , R. C. Gimple , et al., “Three‐Dimensional Bioprinted Glioblastoma Microenvironments Model Cellular Dependencies and Immune Interactions,” Cell Research 30, no. 10 (2020): 833–853. 32499560 10.1038/s41422-020-0338-1 PMC7608409

[302] J. Yi , X. Shi , Z. Xuan , and J. Wu , “Histone Demethylase UTX/KDM6A Enhances Tumor Immune Cell Recruitment, Promotes Differentiation and Suppresses Medulloblastoma,” Cancer Letters 499 (2021): 188–200. 33253789 10.1016/j.canlet.2020.11.031 PMC7779746

[303] T. Kondo , “Glioblastoma‐Initiating Cell Heterogeneity Generated by the Cell‐Of‐Origin, Genetic/Epigenetic Mutation and Microenvironment,” Seminars in Cancer Biology 82 (2022): 176–183. 33453403 10.1016/j.semcancer.2020.12.003

[304] W. Wang , L. Su , Y. Wang , C. Li , F. Ji , and J. Jiao , “Endothelial Cells Mediated by UCP2 Control the Neurogenic‐To‐Astrogenic Neural Stem Cells Fate Switch During Brain Development,” Advanced Science 9, no. 18 (2022): e2105208. 35488517 10.1002/advs.202105208 PMC9218656

[305] T. Zhong , W. Zhang , H. Guo , et al., “The Regulatory and Modulatory Roles of TRP Family Channels in Malignant Tumors and Relevant Therapeutic Strategies,” Acta Pharmaceutica Sinica B 12, no. 4 (2022): 1761–1780. 35847486 10.1016/j.apsb.2021.11.001 PMC9279634

[306] F. Ah‐Pine , M. Khettab , Y. Bedoui , et al., “On the Origin and Development of Glioblastoma: Multifaceted Role of Perivascular Mesenchymal Stromal Cells,” Acta Neuropathologica Communications 11, no. 1 (2023): 104. 37355636 10.1186/s40478-023-01605-x PMC10290416

[307] “Dostarlimab: An Answer for Rectal Cancer?,” Cancer Discovery 12, no. 8 (2022): 1828–1829. 10.1158/2159-8290.CD-NB2022-0047 35771084

[308] K. White , K. Connor , M. Meylan , et al., “Identification, Validation and Biological Characterisation of Novel Glioblastoma Tumour Microenvironment Subtypes: Implications for Precision Immunotherapy,” Annals of Oncology 34, no. 3 (2023): 300–314. 36494005 10.1016/j.annonc.2022.11.008

[309] E. R. Laws and K. Thapar , “Brain Tumors,” CA: A Cancer Journal for Clinicians 43, no. 5 (1993): 263–271. 8364768 10.3322/canjclin.43.5.263

[310] H.‐M. Jeon , J. Y. Kim , H. J. Cho , et al., “Tissue Factor Is a Critical Regulator of Radiation Therapy‐Induced Glioblastoma Remodeling,” Cancer Cell 41, no. 8 (2023): 1480–1497.e9. 37451272 10.1016/j.ccell.2023.06.007 PMC10530238

[311] K. Shimizu , A. Kahramanian , M. A. D. A. Jabbar , et al., “Photodynamic Augmentation of Oncolytic Virus Therapy for Central Nervous System Malignancies,” Cancer Letters 572 (2023): 216363. 37619813 10.1016/j.canlet.2023.216363 PMC10529118

[312] J. Na , S. Shaji , and C. O. Hanemann , “Targeting Histone Deacetylase 6 (HDAC6) to Enhance Radiation Therapy in Meningiomas in a 2D and 3D In Vitro Study,” eBioMedicine 105 (2024): 105211. 38917510 10.1016/j.ebiom.2024.105211 PMC11255518

[313] M.‐D. Ilie , A. Vasiljevic , P. Bertolino , and G. Raverot , “Biological and Therapeutic Implications of the Tumor Microenvironment in Pituitary Adenomas,” Endocrine Reviews 44, no. 2 (2023): 297–311. 36269838 10.1210/endrev/bnac024

[314] N. Yan , W. Xie , D. Wang , et al., “Single‐Cell Transcriptomic Analysis Reveals Tumor Cell Heterogeneity and Immune Microenvironment Features of Pituitary Neuroendocrine Tumors,” Genome Medicine 16, no. 1 (2024): 2. 38167466 10.1186/s13073-023-01267-3 PMC10759356

[315] P. K. Brastianos , A. E. Kim , A. Giobbie‐Hurder , et al., “Phase 2 Study of Pembrolizumab in Patients With Recurrent and Residual High‐Grade Meningiomas,” Nature Communications 13, no. 1 (2022): 1325. 10.1038/s41467-022-29052-7 PMC8921328 35289329

[316] J. Yeung , V. Yaghoobi , D. Miyagishima , et al., “Targeting the CSF1/CSF1R Axis Is a Potential Treatment Strategy for Malignant Meningiomas,” Neuro‐Oncology 23, no. 11 (2021): 1922–1935. 33914067 10.1093/neuonc/noab075 PMC8563319

[317] M.‐D. Ilie , D. de Alcubierre , A. L. Carretti , E. Jouanneau , and G. Raverot , “Therapeutic Targeting of the Pituitary Tumor Microenvironment,” Pharmacology and Therapeutics 250 (2023): 108506. 37562699 10.1016/j.pharmthera.2023.108506

[318] A. Zhang , Y. Xu , H. Xu , et al., “Lactate‐Induced M2 Polarization of Tumor‐Associated Macrophages Promotes the Invasion of Pituitary Adenoma by Secreting CCL17,” Theranostics 11, no. 8 (2021): 3839–3852. 33664865 10.7150/thno.53749 PMC7914368

[319] I. C. Glitza Oliva , G. Schvartsman , and H. Tawbi , “Advances in the Systemic Treatment of Melanoma Brain Metastases,” Annals of Oncology 29, no. 7 (2018): 1509–1520. 29790899 10.1093/annonc/mdy185

[320] T. Ajithkumar , C. Parkinson , K. Fife , P. Corrie , and S. Jefferies , “Evolving Treatment Options for Melanoma Brain Metastases,” Lancet Oncology 16, no. 13 (2015): e486–e497. 26433822 10.1016/S1470-2045(15)00141-2

